# TrackUSF, a novel tool for automated ultrasonic vocalization analysis, reveals modified calls in a rat model of autism

**DOI:** 10.1186/s12915-022-01299-y

**Published:** 2022-07-12

**Authors:** Shai Netser, Guy Nahardiya, Gili Weiss-Dicker, Roei Dadush, Yizhaq Goussha, Shanah Rachel John, Mor Taub, Yuval Werber, Nir Sapir, Yossi Yovel, Hala Harony-Nicolas, Joseph D. Buxbaum, Lior Cohen, Koby Crammer, Shlomo Wagner

**Affiliations:** 1 Sagol Department of Neurobiology, University of Haifa, 3498838 Haifa, Israel; 2 The Integrated Brain and Behavior Research Center (IBBR), Faculty of Natural Sciences, University of Haifa, Mt. Carmel, 3498838 Haifa, Israel; 3 Department of Electrical Engineering, The Technion, 32000 Haifa, Israel; 4 School of Zoology, Faculty of Life-Sciences, Tel-Aviv University, Tel Aviv, Israel; 5 Department of Evolutionary and Environmental Biology and Institute of Evolution, University of Haifa, Haifa, Israel; 6 The Department of Psychiatry and The Seaver Autism Center for Research and Treatment, Icahn School of Medicine at Mount Sinai, New York, NY 10029 USA

**Keywords:** Ultrasonic vocalizations, Computational tool, Behavioral analysis, Animal models, Autism spectrum disorder, Shank3-deficient rats, Bat echolocation, Appetitive calls, Aversive calls, Mating calls

## Abstract

**Background:**

Various mammalian species emit ultrasonic vocalizations (USVs), which reflect their emotional state and mediate social interactions. USVs are usually analyzed by manual or semi-automated methodologies that categorize discrete USVs according to their structure in the frequency-time domains. This laborious analysis hinders the effective use of USVs as a readout for high-throughput analysis of behavioral changes in animals.

**Results:**

Here we present a novel automated open-source tool that utilizes a different approach towards USV analysis, termed TrackUSF. To validate TrackUSF, we analyzed calls from different animal species, namely mice, rats, and bats, recorded in various settings and compared the results with a manual analysis by a trained observer. We found that TrackUSF detected the majority of USVs, with less than 1% of false-positive detections. We then employed TrackUSF to analyze social vocalizations in *Shank3*-deficient rats, a rat model of autism, and revealed that these vocalizations exhibit a spectrum of deviations from appetitive calls towards aversive calls.

**Conclusions:**

TrackUSF is a simple and easy-to-use system that may be used for a high-throughput comparison of ultrasonic vocalizations between groups of animals of any kind in any setting, with no prior assumptions.

**Supplementary Information:**

The online version contains supplementary material available at 10.1186/s12915-022-01299-y.

## Background

Vocal communication is fundamental to the social interactions of many mammalian species [1–3]. In humans, vocal communications are highly dynamic, with distinct vocal signals that are typical of different types of social interactions and distinct emotional states [4, 5]. Mice and rats also emit various vocal signals in diverse social contexts and activities, such as parenting, mating, fighting, and playing [6], which are mostly in the ultrasonic range. Such ultrasonic vocalizations (USVs) reflect the emotional state of the animal and facilitate or inhibit social interaction [7–9]. Although controversial, USVs have gained interest as a proxy model for speech and language [10–12] as well as for affective vocal communication in humans [13, 14]. USVs are also used by mammals for other purposes. For example, in addition to social vocalizations [15], echolocating bats use them for sensing the environment and for prey capturing [16]. Notably, USVs can be easily recorded and monitored across extended periods of time in various contexts and environments, by simply positioning an ultrasonic microphone in the animals’ vicinity. Thus, USVs are a rich and accessible source of information about the behavior and emotional state of various mammals and can be used for screening potential therapeutics in animal models of human pathological conditions [17–19]. Indeed, modified social vocalization activity was previously studied in various mouse models of autism spectrum disorder (ASD), where impairment in social communication is a core symptom [12, 20, 21]. However, the analysis of USVs is usually performed by manual or by semi-automated methodologies which extract discrete USVs from the audio recording and categorize them according to their structure in a spectrogram [22–24]. These highly laborious and observer-dependent methodologies hinder an efficient and large-scale use of such approach for high-throughput analysis of changes in social communication in animal models. During the last decade, several computerized tools for automated or semi-automated detection and categorization of USVs were reported [25–30]. Yet, most of these tools are rather complex and require the user to pre-define many parameters, or to train machine-learning algorithms on large samples of specific types of calls, hence are not easily employed by laboratories that are not specialized in animal vocalizations.

Here we present TrackUSF, an automated, high-throughput, open-source, and easy-to-use tool, which we developed to analyze USVs of any kind with no prior assumptions. This unsupervised analysis tool, which does not necessitate detection and characterization of discrete USVs, requires pre-defining only one parameter (the threshold of signal strength) and does not demand any training of either the system or the user. Notably, TrackUSF aims to compare ultrasonic vocal signals between groups of animals at the frequency domain and does not supply information about USV structure or syntax. We validated the efficacy of TrackUSF by employing it to analyze several types of well-studied forms of animal vocalizations in various settings: mouse mating calls, rat social calls, and bat echolocation calls. We then demonstrated its usefulness for identifying modified social vocalizations in animal models of neurodevelopmental diseases by revealing impaired social communications in adult male *Shank3*-deficient rats, a rat model of autism. Thus, TrackUSF is a simple and easy-to-use system that may be used for a high-throughput comparison of ultrasonic vocalizations between groups of animals of any kind in any setting, with no prior assumptions.

## Results

### TrackUSF

**Fig. 1:**
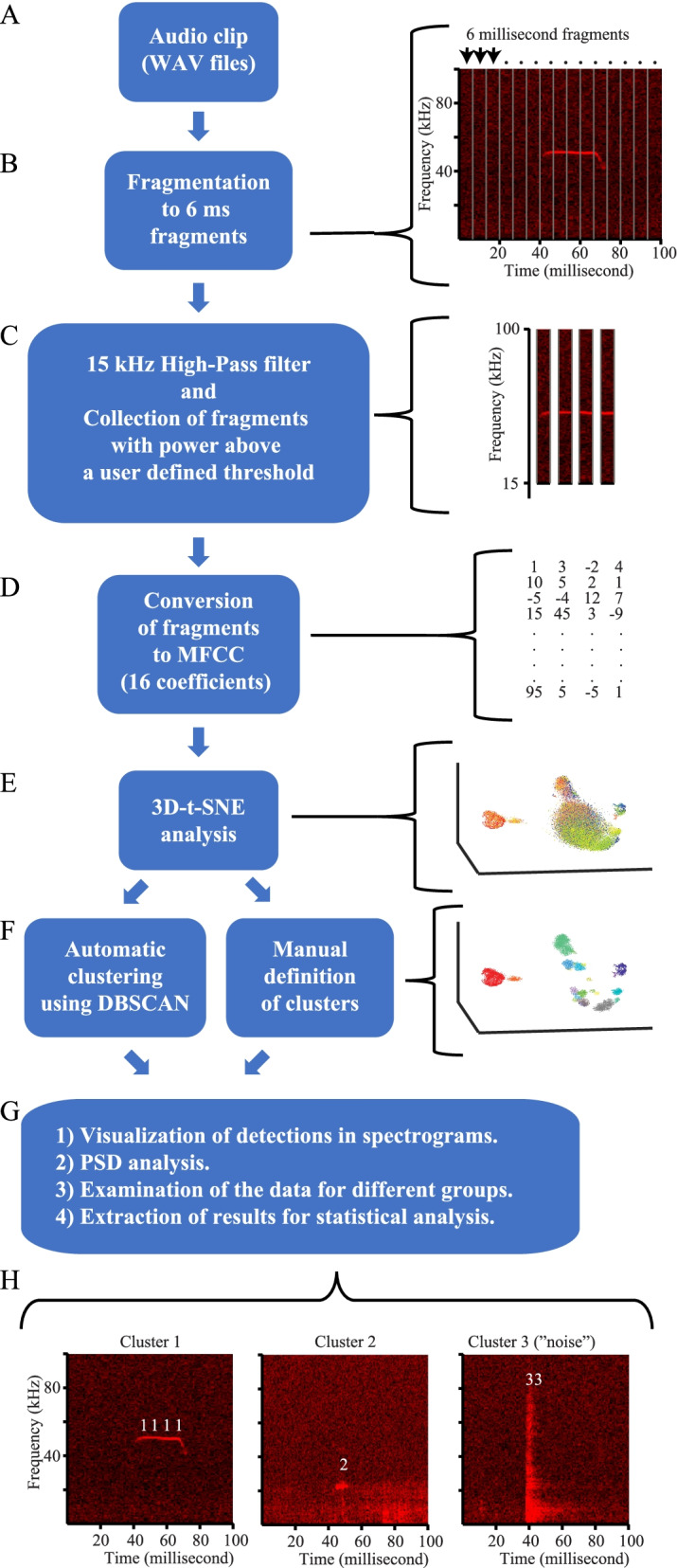
The TrackUSF pipeline. **A** All audio clips of a given experiment are pooled and analyzed together. **B** Each audio clip is divided into 6-ms fragments. **C** All fragments then pass a 15-kHz high-pass filter, and those that contain signals exceeding a predetermined power threshold (USFs) are collected. **D** The power spectrum between 15 and 100 kHz of each USFs is converted to 16 Mel-frequency cepstral coefficients (MFCCs). **E** MFCCs of all USFs are then analyzed together using a 3-dimensional (3D) T-distributed Stochastic Neighbor Embedding (t-SNE) for dimensionality reduction and visualization. **F** Clusters are defined, either manually or using DBSCAN automatic clustering algorithm. **G** Example of further analyses of the results enabled by TrackUSF. **H** Also enabled is the examination of the USFs on the spectrogram of their corresponding audio clip

TrackUSF is designed for high-throughput automated analysis of auditory recordings in the ultrasonic range (20–100 kHz). As depicted in Fig. 1, each auditory clip is divided into 6-ms fragments which are filtered using a 15-kHz high-pass filter. First, all fragments that contain signals exceeding a predetermined power threshold (ultrasonic fragments, USFs herein) are collected. Notably, this threshold is the only parameter that needs to be predetermined by the user. Then, the power spectrum between 15 and 100 kHz of each USFs is converted to 16 Mel-frequency cepstral coefficients (MFCCs). Mel-frequency features represent the short-term power spectrum of a sound, based on a linear cosine transform of a log power spectrum on a nonlinear Mel-scale of frequency, with the frequency bands equally spaced according to the Mel-scale [31, 32]. MFCCs of USFs pooled from all audio clips of the experiment are then analyzed together using a 3-dimensional (3D) T-distributed Stochastic Neighbor Embedding (t-SNE) for visualization of the multi-dimensional dataset. 3D t-SNE models each high-dimensional vector by a point in a three-dimensional space to such a degree that similar vectors are modeled by nearby points, while dissimilar vectors are modeled by distant points with high probability. Following t-SNE analysis, distinct clusters are defined, either manually or using the Density-Based Spatial Clustering of Applications with Noise (DBSCAN) automatic clustering algorithm (see graphical user interface in Additional file 1: Fig. S1). In addition to the t-SNE graphs, the software generates a Matlab file for each audio clip, which contains the time stamps and cluster affiliation of each USF. This enables the software to present the detected USFs on the spectrogram of the audio clip and to analyze the power spectrum density (PSD) of any given combination of clusters and the number of USFs for each cluster. Another type of output is an Excel file detailing the number of USFs of each cluster for each audio clip analyzed.

### Validation of the TrackUSF methodology with mouse mating calls

**Fig. 2:**
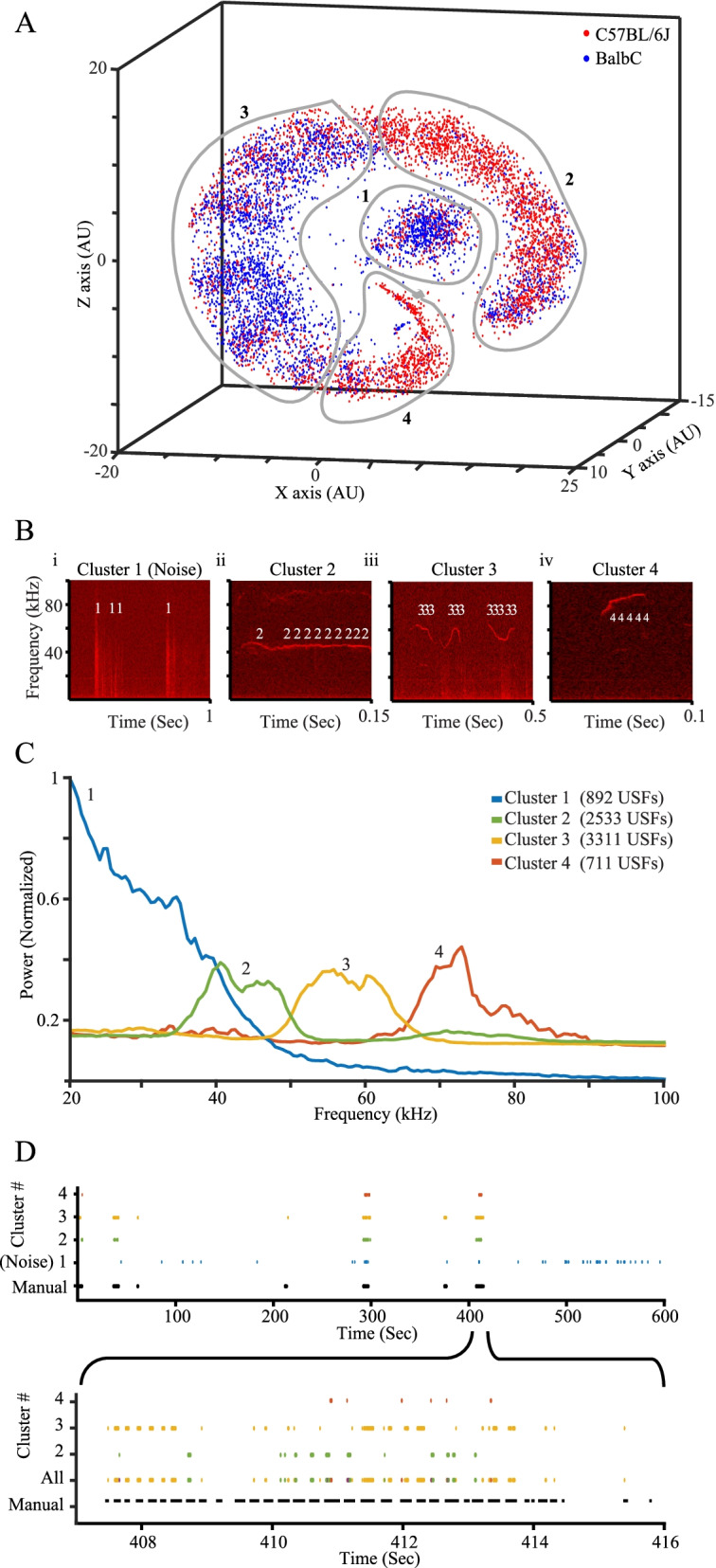
Automated analysis of mouse mating calls using TrackUSF. **A** 3D t-SNE analysis of all USFs recorded from three C57BL/6J and three BalbC male-female pairs. Each USF is represented by a dot, color-coded for the strain. Black numbers represent the distinct clusters, manually defined by the drawn gray lines. Note the clear separation of cluster 1, which includes non-vocal signals defined as noise. **B** Examples of spectrograms showing USFs from all clusters, each marked as the number of the cluster it is linked to, superimposed by the TrackUSF software on their corresponding non-vocal (i) or vocal (ii–iv) signals. **C** PSD analysis of the distinct clusters shown in **A**. The total number of USFs in each cluster is detailed in the legend. Note the unique profile of cluster 1, which is mainly enriched with non-vocal signals (noise). **D** Above: co-localization of USFs (each of which is represented by a single colored dot) and manually defined USVs (black lines, the length of which is proportional to the USV duration) during a whole 10-min-long audio recording of mouse mating calls. Note the various sequences of USVs, separated by prolonged silent periods. Below — one USV sequence displayed in higher resolution, with the co-localized USFs (excluding cluster 1). Note the accurate detection of most USVs by the various types of USFs

To validate TrackUSF, we first compared it to the manual USV-based methodology [6, 33] by analyzing mating calls of C56BL/6J and BalbC mice. It should be noted that hereafter we use the term “call” for any type of ultrasonic vocalization, regardless of its structure or sequence. The USV-based analysis took ~30 work-hours of a well-trained observer, while TrackUSF processed the same data in ~15 min on a standard computer. Figure 2A depicts the t-SNE analysis of all USFs detected by TrackUSF, with each USF represented by a single dot color-coded according to the mouse strain. This analysis revealed segregation between C57BL/6J (red) and BalbC (blue) USFs, suggesting distinct vocalization characteristics. We used the option of manual clustering of TrackUSF to define four clusters of USFs (Fig. 2A, gray lines) based on separation in space and on the distinct segregation of the two strains in each cluster. This enabled us to inspect each USF with respect to its corresponding USV by overlaying detected USFs onto audio-clip spectrograms. As exemplified in Fig. 2B, groups of USFs, denoted by their cluster numbers, overlap distinct USVs. The first example (Fig. 2Bi) included only non-vocal sounds and was enriched with USFs from cluster 1 (Fig. 2A), suggesting that cluster 1 is mostly composed of non-vocal sounds (herein termed noise). Other examples include USVs represented by USFs originating from clusters 2–4 (Fig. 2Bii–iv).

To further analyze each of the clusters we defined, we plotted the PSD profile of USFs from each cluster. As apparent in Fig. 2C, all clusters, except for cluster 1, showed clear, distinct peaks at specific frequencies. In contrast, cluster 1 included USFs of variable frequencies, mainly in the lower range. Given this PSD profile, and our findings that these USFs represent noise, cluster 1 was excluded from all downstream analyses of this dataset.

To compare the results of TrackUSF with those obtained using the manual USV-based methodology, we plotted the distribution of the USFs for each cluster and the manually detected USVs over time. As depicted in Fig. 2D, manually detected USVs appeared in sequences (songs), with prolonged periods of silence between them. Notably, almost all USVs were represented by at least one USF (from clusters 2–4), with no apparent false-positive USFs.

**Fig. 3:**
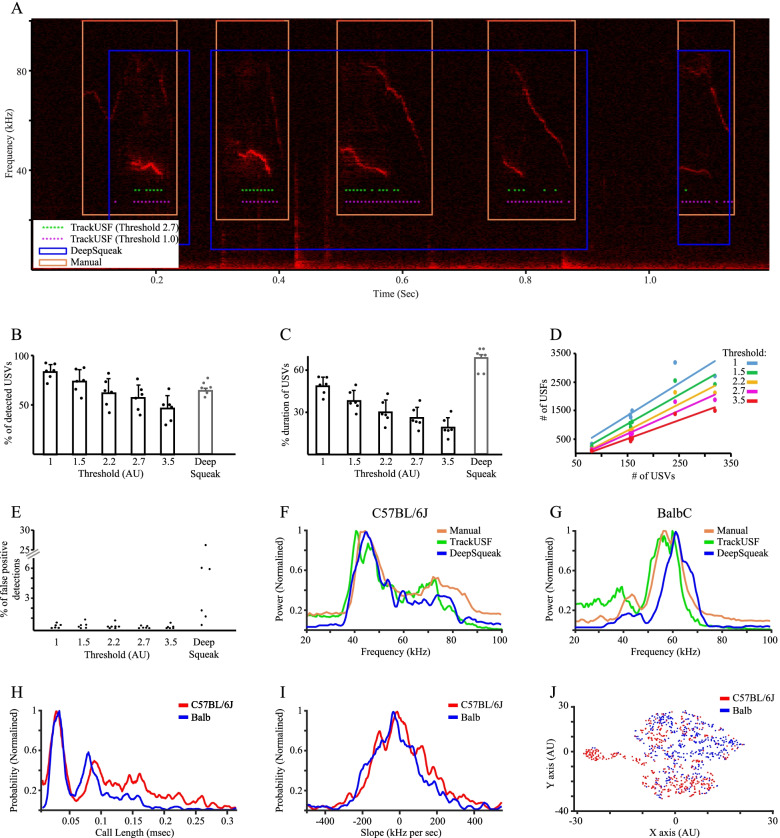
TrackUSF accurately captures the majority of the manually detected USVs and enables their further characterization. **A** An example of a spectrogram showing a sequence of USVs, as defined manually (orange boxes) and by DeepSqueak (blue boxes). USFs detected by TrackUSF at two threshold levels are marked by the green (threshold = 2.7 a.u.) and pink (threshold = 1 a.u.) asterisks. **B** Mean percentage of manually defined USVs, overlapped by at least one USF, using five different thresholds (1, 1.5, 2.2, 2.7, and 3.5 a.u.), as well as by DeepSqueak-defined USVs (gray bar). **C** Mean percentage of coverage of the total duration of manually defined USVs by USFs for each of the various thresholds, as well as by DeepSqueak-defined USVs (gray bar). **D** Number of detected USFs for each audio clip plotted as a function of the number of the corresponding manually detected USVs, for each of the various thresholds. Each dot represents a distinct audio clip analyzed using a distinct threshold. Distinct colors represent distinct thresholds, as depicted in the legend. **E** Percentage of all detected USFs from all clusters, except for cluster 1 (noise), that were found to represent non-USV signals (false-positive detections) for each of the various thresholds, as well as for DeepSqueak. Each point represents a distinct audio clip. **F** Mean PSD profiles of calls emitted by C57BL/6J mice pairs detected either manually (orange), by DeepSqueak (blue), or by TrackUSF (green). The TrackUSF analysis used a threshold of 2.7 a.u., after scaling the PSD curve of each cluster to the number of USFs and summing the curves separately for C57BL/6J mice. **G** As is **F**, for the calls emitted by BalbC mice. **H** Normalized probability function of DeepSqueak-detected USVs according to their duration. **I** Normalized probability function of DeepSqueak-detected USVs according to their slope. **J** K-means clustering of DeepSqueak-detected USVs according to their length, slope, and principal frequency, separately for each mouse strain. All error bars represent SEM

To evaluate the effectiveness of TrackUSF as an analysis tool for ultrasonic vocalizations, we compared its analytical abilities to those of DeepSqueak, one of the most cited (cited by 99 articles, February 3, 2022, Google Scholar) recent computerized tools for such analysis [27]. We set the tonality level of DeepSqueak to 0.15 arbitrary units (a.u.) since the default level of 0.3 a.u. yielded poor results. Analyzing the same dataset described above (Fig. 2) using DeepSqueak took a similar amount of time as using TrackUSF. Figure 3A shows an example of a spectrogram analyzed manually (detected USVs within orange squares), by DeepSqueak (detected USVs within blue squares), and by TrackUSF using two threshold levels (detected USFs marked by asterisks color-coded according to threshold level). As apparent, in this case, DeepSqueak mistakenly defined three distinct USVs as a single one, a mistake that repeated itself multiple times (~10% of detected USVs, see summary in Additional file 2: Table S1). As apparent, TrackUSF detected all USVs in the example by at least one USF. However, detected USFs covered only part of each USV (mainly the areas of stronger signals), in a manner strongly dependent on the threshold level. For a quantitative comparison between the various methods, we employed TrackUSF to analyze the data using five distinct threshold levels (1, 1.5, 2.2, 2.7, and 3.5 a.u.). As expected, the lower the threshold, the longer the time it takes for TrackUSF to analyze the same set of data. In our case, the range was between ~20 min for threshold = 1 to ~10 min for threshold = 3.5. The percent of manually detected USVs which were overlapped by at least one USF varied between 84% in the lowest threshold (1 a.u.) and 46% in the highest threshold (3.5 a.u.), while DeepSqueak performed at 68% (Fig. 3B, Additional file 2: Table S1). The total duration of manually defined USVs that was also occupied by USFs varied between 48% in the lowest threshold and 19% in the highest, while DeepSqueak captured 67% of USV duration (Fig. 3C, Additional file 2: Table S1). This is most likely because many USVs are interrupted by gaps of silence or low amplitude signals, which are counted in their duration by USV-based methodologies but do not contain USFs (see example in Fig. 2Biii).

Nevertheless, we found a statistically significant correlation between the number of manually detected USVs and the number of USFs detected by TrackUSF for all threshold levels (Pearson correlation, *R*^2^=0.81, 0.88, 0.91, 0.92, 0.93, respectively, *p*<0.001 for all; Fig. 3D). We also found that less than <1% of all USFs were false positive (not representing any real USV), regardless of the threshold level (Fig. 3E). In contrast, DeepSqueak had a much higher level of false-positive detections, ranging between 0.3 and 26% for the various audio clips and averaging at 6.9% (Fig. 3E, Additional file 2: Table S1).

To assess the ability of TrackUSF to capture differences in call frequency profiles between animal groups, we compared the PSD analyses of the manually detected USVs and DeepSqueak to those obtained using TrackUSF, separately for the C57BL/6J (Fig. 3F) and BalbC (Fig. 3G) calls. Interestingly, this analysis identified a clear difference between the two strains, with the USVs of C57BL/6J mice showing the main peak at a lower pitch (40 kHz) compared to the higher pitch (60 kHz) of the BalbC USVs. To verify that similar characteristics are identified with TrackUSF (using a threshold of 2.7 a.u.), we scaled the PSD curve of each cluster to the number of USFs in this cluster. We then summed the curves of the scaled clusters separately for C57BL/6J and BalbC mice. This analysis yielded PSD curves that were highly similar to those achieved using the manually extracted USVs (Fig. 3F, G). Notably, a similar analysis of the USVs detected by DeepSqueak from the same recordings yielded very similar PSD profiles for C57Bl/6J mice (Fig. 3F), but was shifted towards higher frequencies compared to manually detected USVs in the case of BalbC mice (Fig. 3G).

Since DeepSqueak enables automatic analysis of two more parameters for the detected USVs, call length and slope, we compared the probability functions of these parameters between the two strains. We found that these parameters do not yield a better separation between the USVs of the two strains than call frequency (Fig. 3H, I). Finally, when employing the ability of DeepSqueak to cluster (using K-means clustering) the detected USVs based on all three parameters (principal frequency, call length, and slope; Fig. 3J), we found that the segregation between the two strains was apparently similar to that achieved using TrackUSF (compare to Fig. 2A), which is based upon signal frequency and amplitude only.

Thus, TrackUSF enables automated and time-efficient analysis of mating calls in mice in a manner that identifies the majority of USVs detected either manually or by DeepSqueak. Accordingly, the number of USFs identified using TrackUSF correlated very well with the number of USVs detected by the USV-based methodologies. Moreover, TrackUSF seems superior over DeepSqueak in its very low false-positive detections and by avoiding joining multiple USVs together. Finally, despite the relatively limited coverage of USV duration by TrackUSF, it accurately captures the spectral characterization of the calls and allows separation between animal groups accordingly.

### Validation of the TrackUSF methodology with rat social calls

**Fig. 4:**
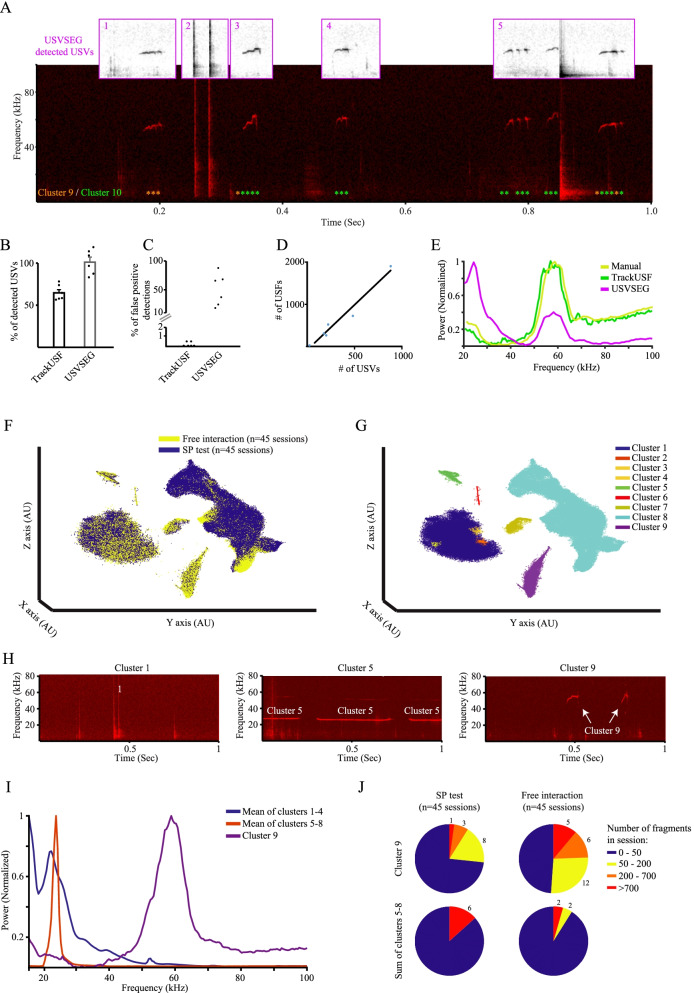
Analyzing rat social vocalizations using TrackUSF. **A** An example of a spectrogram showing a sequence of USVs produced during male-female interactions of SD rats and their detection by USVSEG (purple boxes above). USFs detected by TrackUSF (threshold = 1 a.u.) are marked by the orange (cluster 9) and green (cluster 10) asterisks below. **B** A comparison of the mean percentage of manually detected USV which were automatically detected by either TrackUSF or USVSEG, from 6 audio clips of 10-min SD rat male-female interaction. **C** Percentage of all automatically detected USVs that were found to represent non-USV signals (false-positive detections) for either TrackUSF or USVSEG. Each point represents a distinct audio clip. **D** Number of detected USFs for each audio clip plotted as a function of the number of the corresponding manually detected USVs. Each dot represents a distinct audio clip. **E** Mean PSD profiles of either USVs detected manually (yellow), by USVSEG (pink) or USFs detected by TrackUSF (green). The PSD analysis of TrackUSF detected USFs was conducted after scaling the PSD curve of each cluster to the number of USFs and summing the curves. **F** The t-SNE analysis of all USFs extracted from audio recordings of 45 sessions of free interactions and 45 sessions of SP test, conducted with male SD rats (male-male vocalizations). Each USF is represented by a dot, color-coded for the experimental setting. **G** DBSCAN automatic clustering of the USFs based on the t-SNE analysis result. **H** Examples of spectrograms showing signals composed USFs from three distinct clusters, including noise (cluster 1) or vocalization (clusters 5 and 9) signals. Each spectrogram contains USFs of a single cluster only. **I** Mean PSD profiles of USFs from clusters 1–4 (noise, blue), 5–8 (aversive calls, orange), and 9 (appetitive calls, purple). **J** Pie-charts of the various sessions categorized according to the number of USFs per session, separately for appetitive (cluster 9, upper charts) and aversive (clusters 5–8, lower charts) USFs recorded during SP test (left charts) and free interaction (right charts) sessions

Adult rats emit a relatively high rate of USVs during social (either male-male or male-female) interactions [6, 8, 33, 34]. These calls are generally divided into two categories. The first type is the “22 kHz aversive calls,” which are associated with negative states and aversive situations and are characterized by low pitch (20–30 kHz) and prolonged duration (150–3000 ms). The second type is the “50 kHz appetitive calls,” which are further divided into flat and highly modulated (trills) USVs and are associated with positive states and appetitive situations. These appetitive calls are characterized by high pitch (40–80 kHz) and short duration (10–150 ms). To assess the efficiency of TrackUSF in analyzing rat social vocalizations, we employed this system (using a threshold of 1) for analyzing USVs emitted during 5-min free social interactions between pairs of male and female Sprague Dawley (SD) rats (*n*=6 pairs, one 5-min long audio clip per pair). Similarly to mouse calls (Fig. 2A), TrackUSF analysis of rat calls produced two clear clouds of USFs in the t-SNE analysis: one of noise and one of vocalization fragments (Additional file 1: Fig. S2A). Here we used the automatic clustering option of the TrackUSF software (see the “Methods” section) to define various clusters, displayed in distinct colors in Additional file 1: Fig. S2B. Out of the 10 automatically defined clusters, clusters 1–6 comprised noise fragments, while clusters 7–10 comprised vocalization fragments (see examples in Additional file 1: Fig S2C and Fig. 4A). Accordingly, PSD analysis of the various clusters revealed that clusters 7–10 yielded each a well-defined peak in the range of 35–70 kHz (Additional file 1: Fig. S2D).

We then compared the TrackUSF analysis with the analysis of another recently published computerized tool for segmentation of rodent USVs — USVSEG [29]. We employed this tool to the same dataset of rat calls, using the specific parameters defined by the authors for rat pleasant calls [29]. Figure 4A exemplifies the spectrogram of audio segments defined as USFs by TrackUSF (green and orange asterisks) and those defined as USVs by USVSEG (numbered purple framed above the spectrogram). As apparent, TrackUSF detected all USVs in this spectrogram without including any noise segment. In contrast, besides genuine USVs (frames 1, 3–5), USVSEG also detected several noise segments as USVs (frame 2). Moreover, as happened before to DeepSqueak (Fig. 3A), USVSEG defined multiple USVs as one (frame 5). A quantitative comparison of both tools to manual analysis revealed that TrackUSF detected about 60% of the calls (56.5–78.2% relative to manually detected USVs) while USVSEG detected about 100% of them (in some cases even better than the manual analysis) (Fig. 4B). However, this high rate of detection comes with a price, as USVSEG also detected a very high rate (10–90% relative to manually detected USVs) of noise audio segments as USVs (Fig. 4C). Thus, USVSEG yield a very high rate of false-positive detections. In contrast, TrackUSF made a very low rate of false-positive detections (<1%). As for mice USVs, the number of USFs detected by TrackUSF for rat call was linearly correlated with the number of USVs in each audio clip (Pearson correlation, *R*^2^=0.9618, *p*<0.001; Fig. 4D). Finally, PSD analysis of the audio segments detected as USVs by the manual analysis, Track USF, and USVSEG (Fig. 4E) revealed that TrackUSF yielded a very similar PSD profile as the manual analysis, while USVSEG yielded a rather distinct profile with a significant contribution of noise, as reflected by the prominent peak at low (20–30 kHz) frequency.

For further validation of TrackUSF with rat calls, we used it for analyzing male-male calls in two types of settings: (1) during 5-min long free interactions between an adult (subject) and a juvenile (social stimulus) male rats; (2) during a 5-min social preference (SP) test when the juvenile was located within a triangular chamber at one corner of the arena and investigated by the adult male, as previously described by us [35, 36]. Each subject (*n*=15) was tested for 2–4 sessions in each type of setting. Overall, we recorded audio clips from 45 sessions of free interactions and a similar number of SP tests. Figure 4F depicts the t-SNE analysis of all USFs derived from vocalization recorded in these experiments, color-coded according to the type of session. We used again the automatic clustering option of the TrackUSF software to define various clusters, which are displayed in distinct colors in Fig. 4G. Example spectrograms of vocalization containing USFs of clusters 1, 5, or 9 (each spectrogram contains USFs of a single cluster) are shown in Fig. 4H, while examples representing all other clusters are displayed in Additional file 1: Fig. S2E. As apparent, clusters 1–4 are clearly separate from all other clusters and represent noise. Thus, as was demonstrated above for mouse and rat male-female calls, the noise was readily separated from the vocal signals by TrackUSF. In contrast to clusters 1–4, clusters 5–8 represent vocalizations with the characterization of aversive calls, i.e., prolonged flat vocalizations below 30 kHz, while cluster 9 seems to represent appetitive calls, which are brief and above 50 kHz. This is also apparent from the PSD analysis of the various clusters (Additional file 1: Fig. S2F), shown in Fig. 4I as the mean PSD profiles of clusters 1–4 (noise), clusters 5–8 (aversive calls), and cluster 9 (appetitive calls). Thus, the TrackUSF analysis revealed the same types of aversive, and appetitive vocalizations that are well-known to characterize male-male social interactions in rats. Yet, the clear separation of the aversive calls to several clusters (5–8) suggests small but consistent differences between these calls at the frequency domain, as confirmed by their distinct PSD profiles (Additional file 1: Fig. S2F). The sensitivity of TrackUSF to such changes, especially at the lower range of sound frequency, may be an advantage of TrackUSF over previous techniques.

We further analyzed the numbers of USFs of the various clusters in each session and categorized them according to call type (aversive calls — clusters 5–8; appetitive calls — cluster 9). As shown in Fig. 4J, both types of experimental settings (free interaction and SP test) revealed more sessions enriched with appetitive USFs than sessions enriched with aversive USFs. Also, appetitive USFs are more abundant during free interactions where both animals are free to move in the arena, while aversive USFs are more frequent during SP tests where the juvenile rat is restricted in the triangular chamber. We found a significant difference in abundance between appetitive and aversive USFs in the free interaction setting but not for the SP test, and a significant difference between free interaction and SP test for appetitive USFs but not for aversive calls (Friedman test: *χ*^2^ (3)=28.594, *p*<0.001, post hoc Wilcoxon signed rank test: SP aversive-affiliative: *Z*=−0.994, *p*=0.320; free aversive-affiliative: *Z*=−4.438, *p*<0.001; SP-free affiliative: *Z*=−3.782, *p*<0.001; SP-free aversive: *Z*=−0.013, *p*=0.990). Thus, TrackUSF can be used to capture differences in social vocalization activity between distinct experimental settings.

### Using TrackUSF for analysis of bat calls in a natural setting

**Fig. 5:**
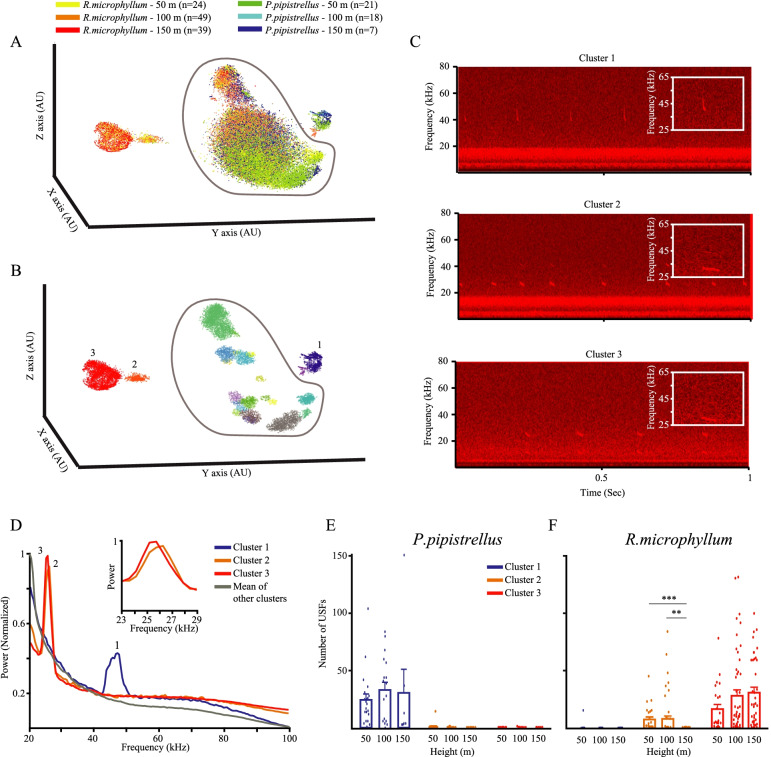
Analyzing bat USVs recorded in a natural scene using TrackUSF. **A** The t-SNE analysis of all USFs extracted from audio recordings of bat calls, made from three distinct heights (50, 100, and 150 m) above ground level at the Hula Valley. These recordings were preselected to include vocalizations of either *R. microphyllum* or *P. pipistrellus*. Each USF is represented by a dot, color-coded according to species and height. Note the gray line manually defining the noise USFs. **B** Automatic clustering of the USFs based on the t-SNE analysis. Note that the cloud of noise USFs defined by the gray line in **A** is now broken into multiple clusters, while only clusters 1–3 represent real vocalizations. **C** Examples of spectrograms of vocalizations represented by USFs of the three clusters. Insets are showing one magnified vocalization per spectrogram. **D** Mean PSD profiles of USFs from the noise clusters (gray), cluster 1 (blue), and clusters 2–3 (red and orange). Inset showing the peaks of clusters 2–3 in higher magnification. **E** Mean number of *P. pipistrellus* USFs detected in each height, separately plotted for the three distinct clusters of USFs. Each dot represents one audio clip. **F** Mean number of *R. microphyllum* USFs detected in each height, separately plotted for the three distinct clusters of USFs. Each dot represents one audio clip. **p<0.01, ***p<0.001, post hoc Dunn test following main effect. All error bars represent SEM

To validate TrackUSF as a mean for analysis of ultrasonic vocalizations of non-rodent animals recorded outside of the lab, we employed it to analyze recordings of bat echolocation calls made in the Hula Valley in Israel, using microphones located at three distinct heights (50 m, 100 m, and 150 m) above ground, hanging on the chord of a large helium balloon. From each height, we manually preselected recordings that contain calls of single bats from two different species: *R. microphyllum* and *P. pipistrellus* (typically 2–4 s long). Figure 5A depicts the t-SNE analysis of all USFs identified in these recordings, color-coded according to the recorded bat species and height of recording (7–49 clips for each case, see legend of Fig. 5A). As apparent, the USFs of different species segregate to distinct clusters. We used the automatic clustering option of the TrackUSF software and observed many small clusters representing noise (circled by a gray line in Fig. 5A, B), which was strong and highly variable between the recordings (Fig. 5C). In addition to the noise, three clusters of vocalization were revealed, of which cluster 1 represented recordings of *P. pipistrellus*, while clusters 2 and 3 mainly represented recordings of *R. microphyllum*. As shown in Fig. 5C, clusters 2 and 3 represent very similar vocalizations, while cluster 1 represents vocalizations with a different distinct structure and frequency. These differences are also observed in the PSD analysis of the three clusters (Fig. 5D), which revealed very similar peaks for clusters 2 and 3 at 25–26 kHz, probably representing two different individuals (see insets in Fig. 5C), while cluster 1 yielded a prominent peak at ~ 47 kHz. We then counted the USFs detected for each cluster at each height and averaged these numbers separately for each bat species. As apparent in Fig. 5E, for *P. pipistrellus*, we found almost only USFs of cluster 1, with no difference in USF number between the various heights (Kruskal-Wallis test: *χ*^2^ (2) = 1.774, *p* = 0.412). In contrast, for *R. microphyllum*, we observed USFs of cluster 3 in all heights (Kruskal-Wallis test: *χ*^2^ (2) = 3.816, *p* = 0.148), while cluster 2 was observed only at 50 m and 100 m, but was absent at 150 m (Kruskal-Wallis test: *χ*^2^ (2) = 18.409, *p* < 0.001). Thus, using TrackUSF, we were able to detect the previously described species-dependent differences in vocalizations [37, 38] without a need to train the system for detecting such differences. Notably, even though the vocalizations composed of USFs from clusters 2 and 3 are almost identical, the use of TrackUSF allowed separating them, showing again its potency in distinguishing between rather similar auditory signals.

### TrackUSF reveals modified social vocalizations of Shank3-deficient rats

**Fig. 6:**
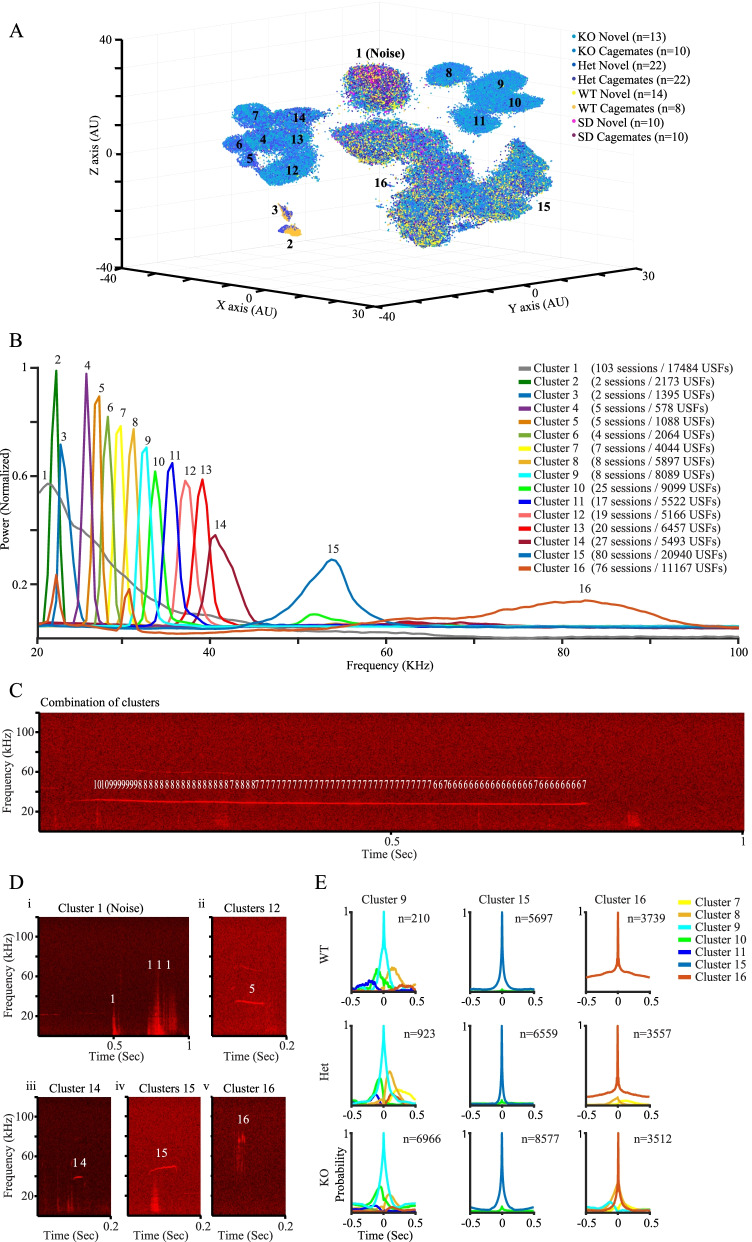
Modified pitch of ultrasonic vocalizations revealed by TrackUSF in *Shank3*-Het and KO. **A** 3D t-SNE analysis of all USFs recorded during all sessions. Each USF is represented by a dot, color-coded for the genotype and familiarity level. Black numbers represent the distinct clusters. Note the clear separation of cluster 1, which included non-vocal signals defined as noise. **B** Mean PSD profiles of all distinct clusters shown in **B**. The number of sessions represented by >10 USFs in each cluster, as well as the total number of USFs of each cluster, are detailed in the figure legend. Note the continuous spectrum in the 25–45-kHz range created by clusters 4–14. **C** Example spectrogram showing USFs from clusters 6–10, each marked as the number of the cluster it is associated with, superimposed by the TrackUSF software on their corresponding single USV. Note the gradual change in cluster number as the USV frequency is getting lower with time. **D** Example spectrograms showing USFs from several clusters, each marked as the number of the cluster it is associated with, superimposed by the TrackUSF software on their corresponding noise (i) or USVs (ii–v) signals. Note the trill-like appearance of USFs from cluster 16 (v). **E** Examples of vicinity curves describing the probability of a USF from any cluster (color-coded for the distinct clusters) to appear before or after USF from a given cluster across the three genotypes of *Shank3*-deficient rats. Note the stability across genotypes exemplified for clusters 9 (left) and 15 (middle), in contrast to the growing tendency of other clusters to combine with cluster 16 in Het and mainly KO animals. It should be noted that USFs of clusters 4–14 were so rare in WT animals that the vicinity curves of cluster 9 are taken from a single WT animal

Following the validation of the TrackUSF methodology, we examined the ability of this system to reveal modified vocalization activity during social interactions in *Shank3*-deficient rats, a rat model of ASD [39, 40]. To record such USVs, we conducted experiments comprised of 10-min-long encounters between dyads of adult male rats of the same genotype, as described for SD rats above (Fig. 4). About half of the experiments comprised encounters between unfamiliar (novel) animals and the other half between familiar animals (cagemates). Besides the three genotypes of *Shank3*-deficient rats (wild-type (WT), heterozygous (Het), and homozygous (KO)), we conducted similar experiments with age-matched adult male SD rats. Overall, we recorded 109 experimental sessions. It should be noted that the relatively larger number of Het sessions (See legend of Fig. 6A) reflects their relative abundance in the litters.

All audio clips were pooled together and analyzed by TrackUSF using a threshold of 2.7 a.u. and clusters were defined manually. As apparent (Fig. 6A), some clusters of USFs (e.g., 15, 16) contained a significant representation of all types of experimental sessions (all genotypes and both familiarity levels). Nonetheless, other clusters (e.g., 4–14) contained almost solely USFs of Het or KO *Shank3*-deficient rats. These results suggested different USVs emitted during social encounters between *Shank3*-deficient rats and their WT littermates or SD rats. To further examine this possibility, we separately analyzed the USFs represented in each cluster by PSD analysis (Fig. 6B).

As with the former datasets described so far, cluster 1, which was clearly separated from all other clusters and included data from all genotypes, comprised USFs of variable frequencies at the lower range. By examining their appearance in the spectrograms, USFs of cluster 1 were found to be non-vocal sounds (noise, see example in Fig. 6Di), and therefore, this cluster was excluded from all further analyses. Clusters 2 and 3 were also excluded from this and other downstream analyses, as they included USFs originating from only two sessions (the number of sessions representing each cluster is detailed in Fig. 6B legend).

Clusters 15 and 16, which showed wide PSD peaks above 50 kHz (Fig. 6B), contained USFs of brief vocalizations that seem to represent the classical 50-kHz appetitive calls (Fig. 3E iv, v), described above for SD rats. In contrast, clusters 4–14, which contained mainly USFs from Het and KO rats displayed relatively sharp, well-defined peaks between 20 and 40 kHz. By their appearance in the spectrograms (Fig. 3D and E ii–iii), USFs of these clusters seem to represent vocalizations which are between the rat classical appetitive and aversive calls. In fact, in many cases, USFs of these clusters (especially 4–10) appeared in sequences along prolonged flat USVs, which resembled classical aversive calls (Fig. 3D).

In order to examine if USFs from the distinct clusters (4–16) tend to appear in certain combinations, we used TrackUSF to calculate their likelihood to appear before or after a USF from a given cluster (hereafter termed “vicinity”), within a time window of 0.5 s for each direction (Fig. 6E, Additional file 1: Fig. S3, color-coded for each cluster). We found that USFs from clusters 4–14 had variable tendencies to appear in certain combinations (see for example Fig. 6E—left panels, for cluster 9), but the highest likelihood was for the repetitive appearance of USFs from the same cluster, as reflected by the high amplitude of their vicinity peak (middle peak in each representative graph in Fig. 6E and Additional file 1: Fig. S3). We termed this likelihood as “repeatability” and further explored it below. In contrast to clusters 4–14, USFs of clusters 15 and 16 showed almost no vicinity with USFs from other clusters in all genotypes (Fig. 6E — middle and right panels).

### Shank3-deficient rats emit higher numbers of low-pitch vocalization fragments

**Fig. 7:**
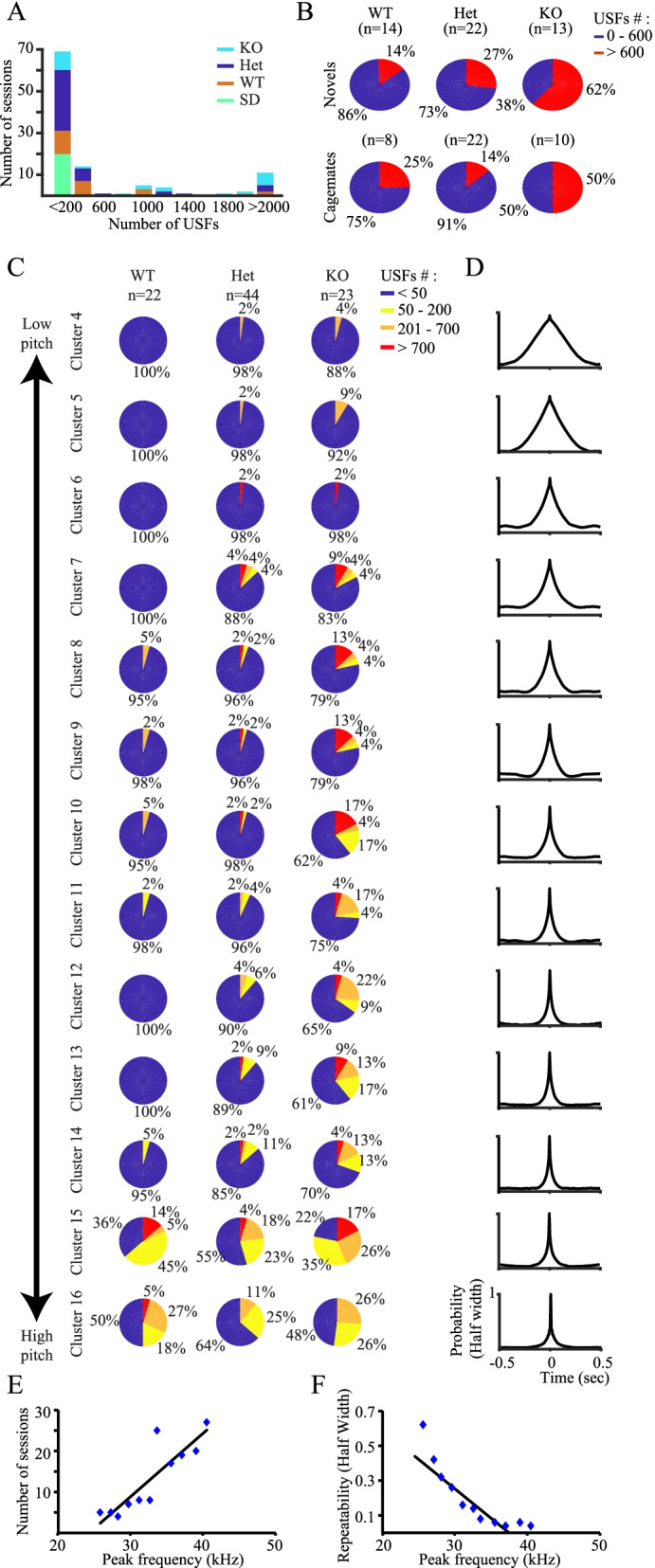
The abundance and repeatability of USFs associated with *Shank3*-Het and KO rats correlate with their pitch. **A** Distributions of the sessions of each of the four genotypes examined based on the total number of USFs (excluding noise) that was detected in each session. **B** Proportions of sessions with more (red) or less (blue) than 600 USFs of all clusters for the Shank3-Het and KO rats and their WT littermates, analyzed separately for dyads of novel animals and cagemates. **C** Proportions of sessions categorized according to the number of USFs emitted during the session, separately calculated for each of clusters 4–16 of Shank3-Het, KO, and WT littermates (combining sessions of novel animals and cagemates). Note the gradual increase in the proportion of sessions with high numbers (>50) of USFs, specifically exhibited by Het and KO animals, for clusters 4–14. **D** Repeatability curves of each of the clusters shown in **C**, combined for Shank3-Het and KO animals. Note the gradual decrease in curve width with cluster number, for clusters of high-pitch USFs. **E** A statistically significant positive correlation was found between the number of sessions contributing to and the peak frequency of each of clusters 4–14, for Shank3-Het and KO rats, combined. **F** A statistically significant negative correlation was found between the half-width of the repeatability curve (shown in **D**) and peak frequency of each of clusters 4–14 for Het and KO animals, combined

We next examined if the number of detected USFs varied between the various genotypes (Fig. 7A). We found that while all SD rats displayed < 200 USFs, the *Shank3*-Het, KO, and WT littermates presented a tri-modal distribution, with many of them displaying > 200 USFs. Nevertheless, only a few sessions of WT rats had > 400 USFs, while most KO sessions had > 600 USFs (Fig. 7A). To further explore this tendency, we categorized each session of the three genotypes of *Shank3*-deficient rats according to the number of detected USFs to low (<600) and high (>600) and examined the proportions of each genotype in these categories separately for cagemates and novel sessions. We found that while WT and Het animals showed a rather similar proportion of 14–27% sessions with > 600 USFs, in KO animals, more than 50% of the sessions were with > 600 USFs (Fig. 7B). This tendency was apparent in all sessions, regardless of the familiarity between the animals (novel animals or cagemates). Statistical analysis revealed a significant difference between the three genotypes (Kruskal-Wallis test: *χ*^2^ (5) = 14.874, *p* = 0.0109), with no familiarity-dependent differences. We therefore combined the two familiarity levels and analyzed the statistical differences between the three genotypes. We found a statistically significant difference between the three genotypes (Kruskal-Wallis test: *χ*^2^ (2) = 12.412, *p* = 0.002). A post hoc analysis revealed a significant difference between KO animals and the two other groups (*p*-adjusted chi-square, KO:Het — *p* = 0.003; KO:WT — *p* = 0.019), with no difference between WT and Het animals.

We further examined this tendency separately for each of the clusters using a slightly more detailed categorization. As shown in Fig. 7C for clusters 4–16, quantities of USFs from clusters 4–14 were differentially distributed between the genotypes. While WT animals showed very restricted numbers of sessions with >50 USFs, KO animals displayed high numbers of such sessions, and Het animals were between WT and KO animals. In contrast, clusters 15 and 16, which seem to represent the 50-kHz appetitive USVs, were similarly distributed between the three distinct genotypes. A closer look into these results suggested a gradient in the number of sessions with >50 USFs between the various clusters, with generally higher numbers of sessions for clusters representing high-pitch calls (Fig. 7C). In agreement with this observation, we found a statistically significant positive correlation (Pearson correlation, *R*^2^=0.77, *p* < 0.0001) between the number of sessions contributing > 10 USFs for a given cluster and the PSD peak frequency of this cluster (Fig. 7E), with high-pitch clusters having more sessions than low-pitch clusters. These results suggest that high-pitch calls are more common among the various sessions.

We also noticed a similar gradient when calculating the probability of USFs to follow or precede other USFs of the same cluster (repeatability) (Fig. 7D). We therefore calculated the half-width of this repeatability curve for each cluster and used it as a proxy for the duration of USVs composed of repeated appearances of the same USF. This analysis was done for Het and KO animals together, as they showed very similar repeatability curves (Additional file 1: Fig. S4), while for WT animals we did not have enough calls to perform such analysis for all clusters. A statistically significant negative correlation (Pearson correlation, *R*^2^=0.78, *p*<0.0001) was found between the PSD peak frequency and repeatability half-width of each cluster (Fig. 7F). Given that wider repeatability half-width is pointing to a longer duration of USVs, this correlation suggests that longer USVs are composed of low-pitch USFs. Taken together, these results suggest that *Shank3*-deficient animals (Het and KO animals) exhibit a spectrum of modified social vocalizations. Within this spectrum, a stronger modification, exhibited by fewer animals, is reflected by calls that are closer to 22-kHz USVs in both their pitch (low) and duration (extended), while weaker and more common levels of modification are reflected by USVs that are closer to 50-kHz calls in both pitch and duration.

## Discussion

During recent years, multiple computerized tools for automated or semi-automated detection and categorization of USVs were presented (see the “Background” section for references). Nevertheless, most of these analysis tools are supervised (for example, XBAT, VoICE, MUPET) and hence require defining a significant number of parameters by a trained user. The unsupervised tools (for example, DeepSqueak) require training of the system to the specific type of vocalizations, thus demanding a manually prepared large dataset to train the system on. Importantly, all these tools focus on the detection of previously recognized types of USVs, categorizing them according to their highly variable structure and analyzing their sequences. Therefore, while these tools may be very useful for researchers studying the information directly encoded by the structure and sequences of social vocalizations [41], they are less suitable as a measure for identifying changes and differences in social communication, some of which may significantly differ from the commonly encountered USVs. Identification of such changes does not necessarily demand analysis of USV complex structure and sequences. Instead, it requires a simple, efficient, and automated methodology for a high-throughput comparison of social vocalizations between groups of animals. The requirement from such methodology is that it would allow good enough sampling of the vocal signals to enable their accurate characterization and trustful comparison of these characteristics between the various animal groups.

Here we presented a novel approach for such comparisons that fits any type of vocalizations in the range of 20–100 kHz, with no prior assumption. Instead of direct identification and analysis of discrete USVs, as used by previous tools, we analyze the frequency-power representation of vocalization activity in a high-throughput manner. To this end, we adopted a methodology commonly used for human speech detection [31, 32, 42, 43] and embedded it in our software. The graphical user interface (GUI) of the open-source TrackUSF software enables loading a large number of audio clips and their sementation to small fragments (USFs) for analysis. Following the t-SNE analysis, TrackUSF enables USF clustering either by an automated algorithm (DBSCAN) or using visually guided manual definition, or a combination of both, which adds to its friendly and flexible use. Following clustering, TrackUSF allows further analyses, such as PSD analysis, as well as the examination of USFs from any combination of clusters by their overlay on the spectrogram of a given audio clip. By analyzing short USFs instead of discrete USVs, TrackUSF gains independence with regard to the USV structure and sequence. This allows TrackUSF to be used for the analysis of any type of ultrasonic vocalization, with no prior assumption regarding its structure and sequence. Moreover, one can use TrackUSF for analysis of any dataset of any size, without a requirement of training the software. Also, using TrackUSF demands only one pre-defined parameter, which is the threshold used for filtering out “silent” fragments. These features make TrackUSF a highly versatile tool that can be easily adopted for various applications by researchers who are not specialized in analyzing ultrasonic vocalizations, for both laboratory and field studies. On the other hand, TrackUSF loses the temporal information encoded by USV structure and sequence, hence may be less suitable for researchers investigating these aspects of vocalizations.

We tested the efficiency of TrackUSF by analyzing three distinct sets of vocalization types: mouse mating calls and rat social calls recorded in the laboratory and bat echolocation calls recorded in the wild. Notably, each of these vocalization types was recorded using a different ultrasonic microphone (see the “Methods” section). In all cases (Figs. 2A, 4F, and 5A), we found that USFs representing non-vocal noise appeared in well-defined clusters, easily separated from all other USFs, which enabled their automated exclusion from further analyses. The ability of TrackUSF to automatically and efficiently separate noise from vocalizations is a substantial advantage over previous methods and spares the tedious manual steps of de-noising, which are critical for analyzing these types of datasets. This feature is most practical for recordings in noisy natural settings, where noise type and level are constantly changing, as exemplified here by the analysis of bat calls in their natural habitat (Fig. 5C).

We found that aside from the noise clusters, USFs of other clusters represent genuine USVs of various types, with distinct clusters representing mainly USFs of different frequencies (Figs. 2C, 4I, and 5D).

By comparing TrackUSF analysis of mouse and rat mating calls with either manual analysis or automated analysis with two recent computerized USV-based tools (DeepSqueak and USVSEG), we found that in both cases TrackUSF represented most manually defined USVs with at least one USF and that the percentage of identified USVs got higher with lowering the threshold and was comparable to the percentage of USVs detected by DeepSqueak. However, the coverage of USV duration by TrackUSF-generated USFs was significantly lower than both computerized tools, which is one limitation of our system. This is partially due to USVs breaking to small fragments by TrackUSF, which left low-level segments in the middle of many USVs uncovered. On the other hand, TrackUSF yielded a very low level of false-positive detections (<1% in all threshold levels), while DeepSqueak generated on average 6.9% false-positive detections and USVSEG yielded very high levels of 10–100%. We conclude that while TrackUSF only partially covers USVs, it samples them good enough and very accurately. Accordingly, we found an excellent match between the PSD analyses of manually detected USVs and TrackUSF fragments, which was slightly better than DeepSqueak and much better than USVSEG. Moreover, the number of TrackUSF-generated USFs was in lineal correlation with the number of manually detected USVs. Altogether, these assessments suggest that TrackUSF may be used for accurate characterization of USVs and their comparison between animal groups at the quantity and frequency domains.

To demonstrate the usefulness of TrackUSF for detecting modified social vocalizations in animal models of pathological conditions, we employed it to study social vocalizations during male-male interactions in *Shank3*-deficient rats [40]. These rats were previously reported to exhibit impaired social approach behavior following playback of appetitive ultrasonic vocalization [44]. TrackUSF revealed a significant number of clusters (4–14) that were mostly enriched with USFs generated by Het and KO animals (Fig. 6A). These calls could not be simply identified as 22-kHz aversive calls, associated with aggressive behavior [18, 45–47], as their PSD peaks created a rather continuous spectrum between 25 and 45 kHz (Fig. 6B) and their duration varied widely from brief (few tens of milliseconds; Fig. 6D) to extended (several hundreds of milliseconds; Fig. 6C).

Interestingly, the PSD peak frequency of clusters 4–14 was found to be negatively correlated with the repeatability index, which serves to estimate the duration of the underlying USVs, and positively correlated with the number of sessions contributing USFs to these clusters (Fig. 7B). Thus, during male-male interactions, *Shank3*-deficient rats seem to make enhanced use in a spectrum of vocalizations, leaning towards lower frequencies and longer durations (22-kHz-like calls). Such calls are rare in WT animals (Fig. 7A) and absent from vocalizations made by SD rats (Fig. 4D). Notably, in a recently published study, we have used TrackUSF to reveal a deficit in mating calls exhibited by Iqsec2 A350V mice, a novel model of ASD. Thus, the modified vocalization revealed by TrackUSF may serve as a convenient and unique readout of modified behavior of various ASD animal models, which may be used to efficiently probe the effect of possible treatments on it.

## Conclusions

We presented a novel open-source computerized tool termed TrackUSF that enables automated high-throughput analysis of ultrasonic vocalization activity. This tool, which avoids analyzing discrete USVs, is mainly suited for detecting differences in any type of ultrasonic vocalization activity between groups of animals. Since TrackUSF does not require any training and demands pre-definition of a single parameter only, it is easily usable by students and investigators who are not specialists in social vocalizations but rather would like to use it as a behavioral readout. We believe that this tool would enable large-scale analysis of modified social vocalization activity in animal models of pathological conditions.

## Methods

### Animals

#### Mice

BalbC and C57BL/6J animals were bred in clean plastic chambers (GM500, Tecniplast, Italy) at 22°C and a 12-h light/12-h dark cycle (light on at 7 am) and received food and water ad libitum.

#### Rats

Subjects were naive Sprague Dawley (SD) male and female rats (8–12 weeks), commercially obtained (Envigo, Israel), and housed in groups of three to five animals per cage. *Shank3*-deficient rats (RRID:RGD_41404705) were a generous gift by Dr Joseph Buxbaum at the Icahn School of Medicine at Mount Sinai. They were bred in a local colony and housed under the same condition described above. Wild-type (WT), heterozygous (Het), and knock-out (KO) littermates were offspring of heterozygous mating pairs. All rats were kept on a 12-h light/12-h dark cycle, light on at 9 pm, with ad libitum access to food and water.

All of the cages contained standard wood chip bedding and cotton wool bedding material. Behavioral experiments took place during the dark phase under dim red light. All experiments were approved by the Institutional Animal Care and Use Committee (IACUC) of the University of Haifa. No animal was excluded from the analysis.

#### Bats

Audio sampling was conducted during September–November 2019 at the Hula Research Center in the upper Galilee, Israel (33° 06′ 47.1″ N 35° 35′ 08.6″ E). An hour before sunset, a tethered 12-m^3^ helium-filled Kingfisher aerostat (LITAS, Jacksonville, FL, USA) was launched to a height of 160 m above ground. Sessions were authorized by Israel’s civil aviation authority. Three “Song Meter SM4BAT FS” bioacoustics recorders (Wildlife Acoustics) were tied to the tether at heights of 50, 100, and 150 m above ground. The recordings were performed at a sampling rate of 192 kHz (converted to 250 kHz for TrackUSF analysis), aiming to document any activity of *R. microphyllum* and *P. pipistrellus* within the vicinity of the aerostat from ground level to roughly 200 m. Peak activity times of *R. microphyllum* and *P. Pipistrellus* in the research area were noted from an hour before to 2 h after sunset. Accordingly, the aerostat was deployed during this time window.

### Experiments

#### Mice

Vocal communications were recorded using a 1/4-inch microphone (Type 4939-A-011), connected to a preamplifier (Type 2670) and an amplifier (Type 2690-0S1, Bruel & Kjaer) in a custom-built sound-shielded box. Vocalizations were sampled at 250 kHz with a CED Micro 1401-3 recording device (Cambridge Electronic Design Limited, Sunnyvale, CA).

In each session, a pair of mice were kept in their home cage and the cage was placed within a custom soundproof box to minimize background noise. The microphone, inserted through the soundproof box lid, was suspended just above the home cage. The system was programmed to record 10 min every hour for 12 h that started after the first encounter between the animals. The audio clips were then analyzed offline in two stages (see below). Collectively, we recorded from 6 mouse pairs. For each of these pairs, one clip that comprised the highest amount of vocalizations among all other clips was used for the comparison with the data obtained by our TrackUSF software.

#### Rats

The experimental setup consisted of a black Plexiglass arena (50 × 50 × 40 cm) placed in the middle of an acoustic chamber (90 × 60 × 85 cm). A computer-connected high-quality monochromatic camera (Flea3 USB3, Point Grey), equipped with a wide-angle lens (Fujinon 6mm fixed focal length C-mount lens, Point Grey), was placed at the top of the acoustic chamber, enabling a clear view and recording of the rat’s behavior using a commercial software (FlyCapture2, Point Grey). Video recordings were carried out at a rate of 30 frames per second. Ultrasonic vocalizations were recorded using a condenser ultrasound microphone (CM16/CMPA, Avisoft) placed high enough above the experimental arena, so the receiving angle of the microphone can cover the whole arena. The microphone was connected to an ultrasound recording interface (UltraSoundGate 116Hme, Avisoft), which was plugged into a computer equipped with the recording software Avi-soft Recorder USG (sampling frequency: 250 kHz; 16-bit format).

#### Bats

Audio recordings were manually analyzed using the Avisoft SASLab software (Avisoft Bioacoustic, Germany). In order to identify the different bat species, the terminal frequency of recorded echolocation signals was measured on screen with a cursor, from the spectrogram (FFT 256 window length, with a Flat Top window). The two species reported here use very distinct call frequencies, so there is no difficulty identifying them: *R. microphyllum* emits multi-harmonic, quasi-constant frequency calls with most energy in the second harmonic around 25–28 kHz while *P. pipistrellus* uses wideband FM calls with terminal frequencies ranging between 45 and 48 kHz [38, 48].

### Audio analysis

#### Manual analysis of mouse mating calls

Audio clips were analyzed offline in two steps. In the first step, single syllables, defined as a discrete USV element separated from other single USV elements by at least 55 ms [49], were manually determined on the spectrograms by a trained observer and fully extracted from the spectrograms. This allowed us to separate the audio files into two vectors: one containing emitted vocalizations with background noise and the second containing the inter-syllable segments which contained only the background noise. Next, we performed a fast Fourier transform (fft) on both vectors and subtracted the later vector from the former to produce a cleaner PSD vector that was used for spectral analysis. All vectors of ultrasonic vocalizations were normalized to the peak value in the range of 30–100 kHz. Thereafter, the second step of the analysis was set to determine the spectral and temporal characteristics of each syllable in order to evaluate the vocal repertoire of the recorded mice.

#### Manual analysis of rat male-female calls

USVs recorded during 10-min sessions of free interaction between male-female couples of SD rats were manually analyzed using our previously published computerized tool HybridMouse [50]. Audio clips were loaded to the GUI of the Matlab-based software and a blind trained observer labeled manually the USVs on the presented spectrogram. This yielded a vector containing the emitted vocalizations for each audio clip. Next, we performed a fast Fourier transform (fft) on this vector to produce a PSD profile that was used for spectral analysis. All vectors of ultrasonic vocalizations were normalized to the peak value in the range of 20–100 kHz.

### TrackUSF

#### Algorithm

Mel-frequency features represent the short-term power spectrum of a sound based on a linear cosine transform of a log power spectrum on a nonlinear Mel-scale of frequency. In this method, the frequency bands are equally spaced according to the Mel-scale [31, 32, 42, 43]. In our study, we expanded the Mel-frequency Cepstrum approach to represent ultrasonic vocalizations using the Matlab function “mfcc” (https://www.mathworks.com/matlabcentral/fileexchange/32849-htk-mfcc-matlab). We changed the lower and upper frequency limits to be between 15 and 100 kHz, with the number of cepstral coefficients enlarged respectively to 16. To analyze these features, we used dimensionality reduction by employing the t-Distributed Stochastic Neighbor Embedding (t-SNE) algorithm [51], an algorithm that is particularly efficient for the visualization of high-dimensional datasets. 3D t-SNE models each high-dimensional matrix by a point in a three dimensions space to such a degree that similar vectors are modeled by nearby points, while dissimilar vectors are modeled by distant points with high probability. In our analysis, we used the function “tsne” in Matlab with the default algorithm “barneshut” and perplexity of 500 points.

The algorithm was embedded into a graphical user interface (GUI) written in Matlab (Additional file 1: Fig. S1 and Additional file 3: “TrackUSF user manual”). Through the GUI, the user chooses sets of audio files for analysis (WAV format), divided according to their group identity. The analysis is then run and outputs the MFCC analyzed data, the t-SNE analyzed data, and the audio files in a Matlab format (“.mat”). Next, the user can open those files for visualization of the data in 3D and for manually defining clusters on top of the t-SNE image result. A second option is to use the Density-Based Spatial Clustering of Applications with Noise (DBSCAN) algorithm or to use a combination of both manual and automatic methods. In our analysis, we used the function “DBSCAN” in Matlab with the threshold for a neighborhood search radius (epsilon) set to 1.5 and the minimum number of neighbors (minpts) set to 50. After defining the clusters, the user can present the detected USFs on the spectrograms of the original data.

All variations of the software and its manual are deposited in GitHub under the following links: https://github.com/shainetser/TrackUSF.

Spectrograms were computed using the standard “spectrogram” function with a window of 512 samples, 50% overlap, and a sample rate of 250kHz. Power spectral density (PSD) for the different clusters was performed using a short-time Fourier transform with the same parameters as for the spectrograms. First, PSDs were performed for each USF separately. Then, the mean PSD for each cluster was calculated by averaging the PSDs of all USFs from the same cluster.

Calculating the probability of USF occurrence relative to USFs from their own cluster or from other clusters was done in a time window of 6 ms for 0.5 s before and after each USF detection. The time window of 6 ms was chosen after examining several other time windows, which gave poorer results.

### Statistics

Data are presented as the mean ± SEM unless otherwise noted. Differences in the means of three or more groups were tested using analysis of variance (ANOVA) followed by Bonferroni post hoc tests, when a significant main effect was found. In case of violation of ANOVA model assumptions (including lack of normal distributions), the Kruskal-Wallis test was performed for comparing distributions of the groups, followed by a post hoc Dunn test with Bonferroni’s adjustment, when a significant result was found. In the case of paired comparison between groups, the Friedman test was used, followed by a Wilcoxon signed ranks test post hoc. When using a cutoff based on biological assumption, a chi-square test was performed. When significant results were obtained, additional chi-square tests were performed between each per of groups, adjusted by Bonferroni’s correction.

## Supplementary Information

**Additional file 1: Figure S1.** The Graphical User Interface (GUI) of the Matlab-based TrackUSF software. **Figure S2.** Examples and PSD profiles of various rats USF clusters. **Figure S3.** Vicinity curves for clusters 4-16, across the three genotypes of Shank3-deficient rats. **Figure S4.** Repeatability curves of clusters 4-16, for WT, Het and KO dyads.

**Additional file 2: Table S1.** Summary of DeepSqueak performance.

**Additional file 3.** TrackUSF Manual.

## Data Availability

All variations of the software are deposited in GitHub under the following links: https://github.com/shainetser/TrackUSF. The datasets used and/or analyzed during the current study are deposited in Mendeley Data and available using the following reference: Netser, Shai (2022), “Data for paper - TrackUSF, a novel tool for automated ultrasonic vocalization analysis, reveals modified calls in a rat model of autism”, Mendeley Data, V2, doi: 10.17632/8d4yz5fyhy.2
