# Supplementary material for: TrackUSF, a novel tool for automated ultrasonic vocalization analysis, reveals modified calls in a rat model of autism
Source: BMC Biol. 2022 Jul 12;20:159. doi: 10.1186/s12915-022-01299-y (PMC9277954; doi:10.1186/s12915-022-01299-y)

Additional file 1: Figures S1-S4

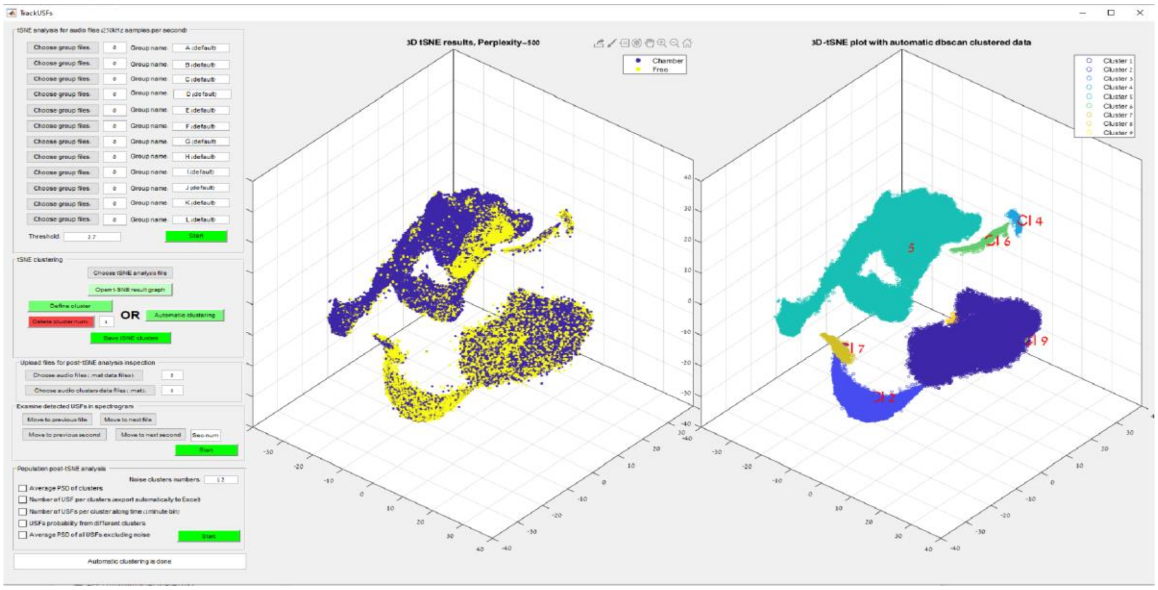

Figure S1: The Graphical User Interface (GUI) of the Matlab-based TrackUSF software.

The left sub-plot shows the t-SNE results color-coded by animal group while the right sub-plot shows the same results color-coded by cluster.

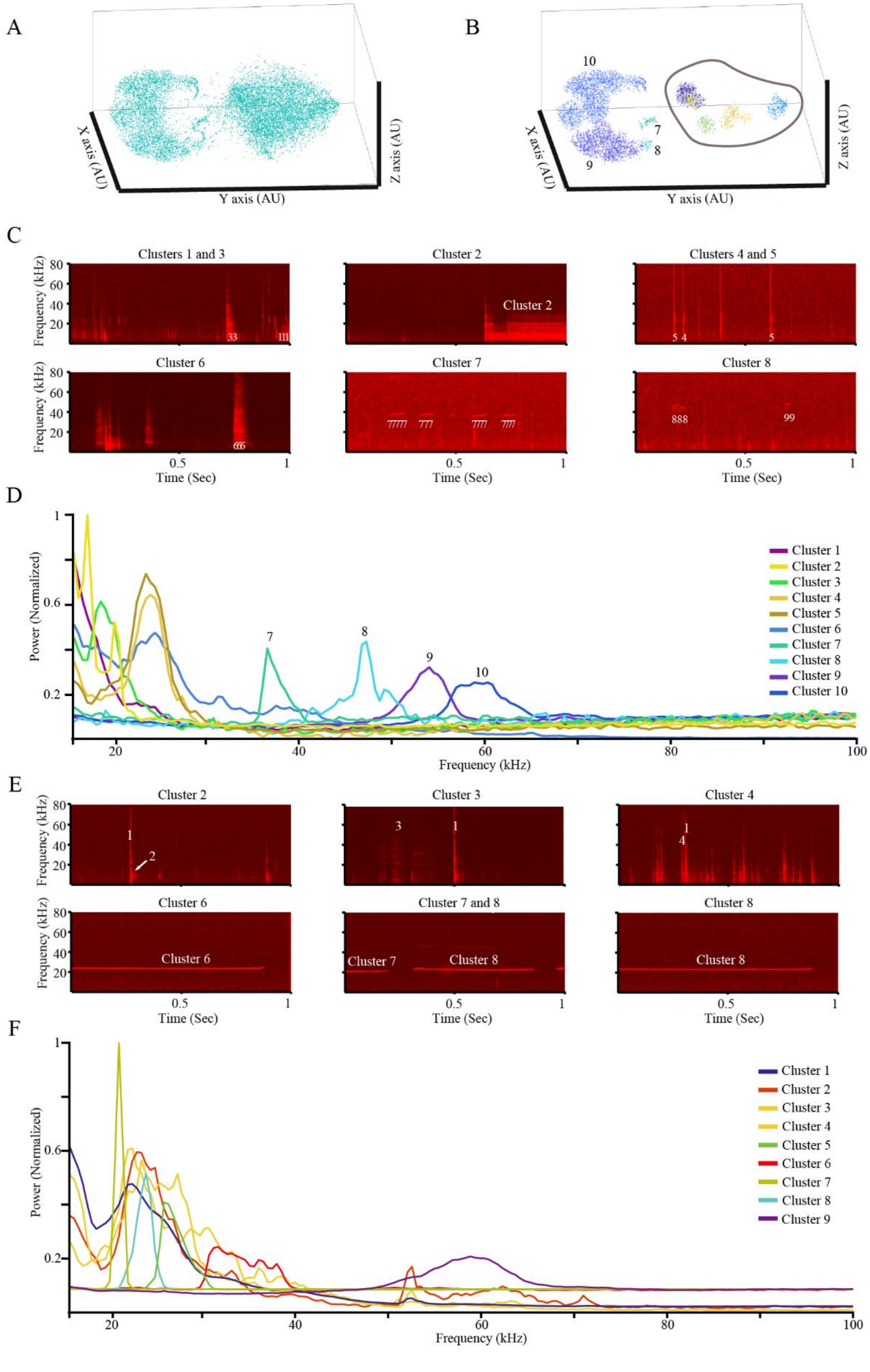

Figure S2. Examples and PSD profiles of various rats USF clusters

A) The 3D t-SNE analysis of all USFs extracted from six 10-min long audio recordings of SD rats male-female free interaction. Each USF is represented by a dot. Note the clear separation between noise (right cloud) and vocalization (left cloud) USFs.

B) DBSCAN automatic clustering of the USFs shown in A, based on the t-SNE analysis result. Each cluster is represented by a distinct color and number. Noise clusters are circled by a dark line.

C) Example spectrograms containing USFs of some clusters, including several noise (1-6) and two vocalizations (7-9) clusters.

D) Mean PSD profiles of all the clusters of USFs detected in the t-SNE shown in B, normalized to the peak of cluster 2 and color coded according to cluster number. Note the clear single peak at frequencies higher than 30 kHz, for clusters of genuine vocalizations (7-10), as compared to the low frequencies of noise clusters (1-6).

E) Example spectrograms containing USFs of the various clusters (besides clusters 1, 5 and 9 which are shown in Fig. 4H) automatically defined by TrackUSF from rat calls during male-male interactions (Fig, 4G). Note that clusters 2-4 (upper panels) represent noise while clusters 6-8 (lower panels) represent aversive calls.

F) Mean PSD profiles of all the clusters of USFs detected in the rat male-male calls (Fig. 4G), normalized to the peak of cluster 7.

Figure S3: Vicinity curves

Vicinity curves for clusters 4-16, across the three genotypes (WT-left, Het-middle and KO-right) of Shank3-deficient rats. In the case of WT animals, clusters 4-14 did not have enough representation for such analysis.

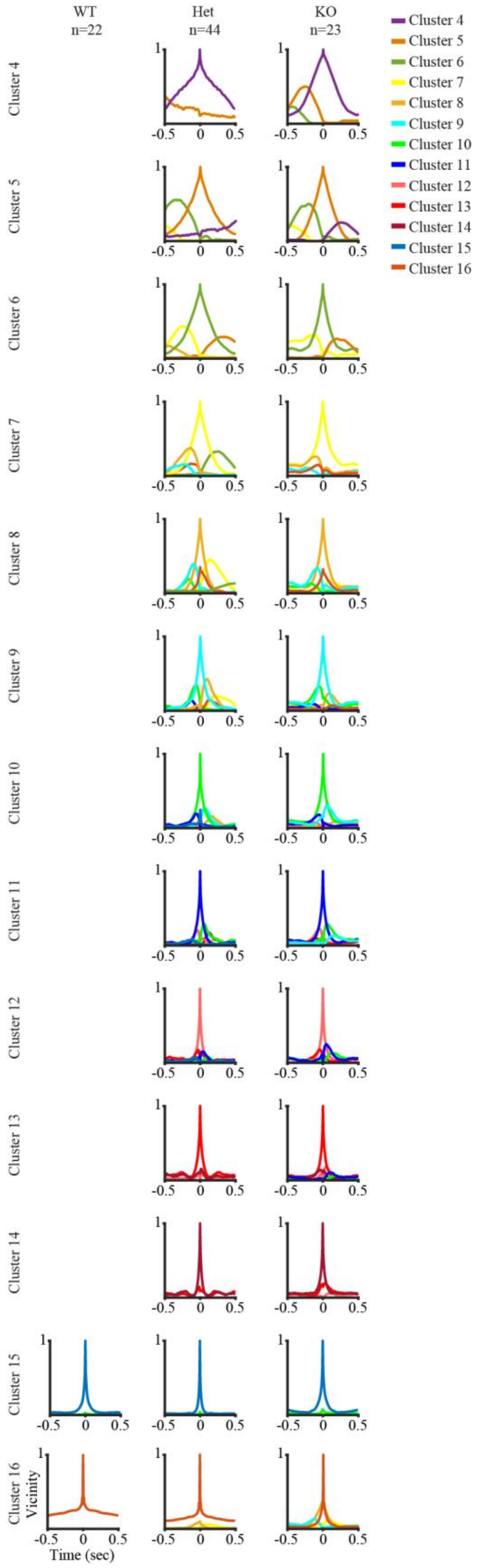

Figure S4: Repeatability curves

Repeatability curves of clusters 4-16, for WT (left), Het (middle) and KO (right) dyads. In the case of WT animals, clusters 4-14 did not have enough representation for such analysis.

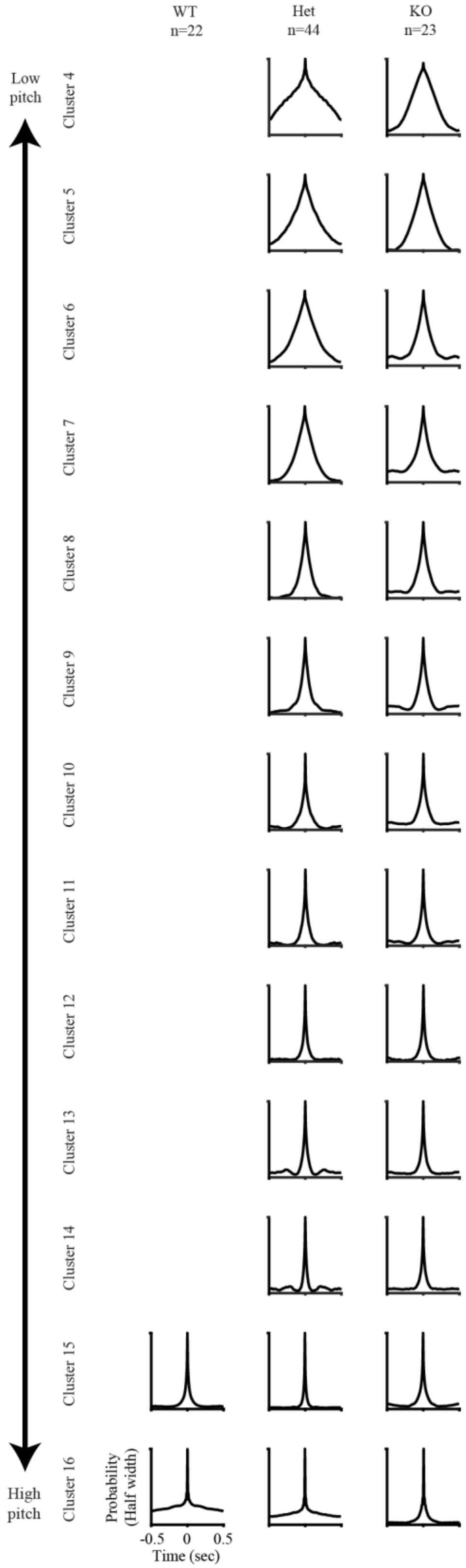

Supplement: Supplementary file 1 — Additional file 1: Figure S1. The Graphical User Interface (GUI) of the Matlab-based TrackUSF software. Figure S2. Examples and PSD profiles of various rats USF clusters. Figure S3. Vicinity curves for clusters 4-16, across the three genotypes of Shank3-deficient rats. Figure S4. Repeatability curves of clusters 4-16, for WT, Het and KO dyads. [file 12915_2022_1299_MOESM1_ESM.pdf]
