# Supplementary material for: TrackUSF, a novel tool for automated ultrasonic vocalization analysis, reveals modified calls in a rat model of autism
Source: BMC Biol. 2022 Jul 12;20:159. doi: 10.1186/s12915-022-01299-y (PMC9277954; doi:10.1186/s12915-022-01299-y)
Supplement: Supplementary file 2 — Additional file 2: Table S1. Summary of DeepSqueak performance. [file 12915_2022_1299_MOESM2_ESM.pdf]

Additional file 2: Table S1: Summary of DeepSqueak performance

Variable	Balb/C pair1	Balb/C pair2	Balb/C pair3	C57 pair1	C57 pair2	C57 pair3	Mean for all sessions
Multiple syllables number	4	12	11	8	14	17	11
False negative number (missed syllables)	32	51	107	42	59	112	67.16
False positive number (noise)	22	11	1	11	3	3	8.5
Partial syllable number	0	28	16	27	17	37	20.83
True positive number (whole syllable)	52	103	206	114	92	112	113.16
Total duration of the detected syllables by Deep squeak (Sec)	3.56	12.72	20.39	13.08	8.19	19.56	12.91
Total duration of manually labeled syllables (Sec)	4.74	17.93	28.38	17.42	14.27	34.08	19.47
**% of Duration**	75.12	70.92	71.83	75.10	57.40	57.40	67.96
**% of False Positives**	26.19	6.04	0.30	6.01	1.79	1.15	6.91
**% of Detected USVs**	61.90	71.98	67.48	77.05	64.88	57.09	66.73

$$\textbf{\% of Duration} = 100 * \frac{\text{DS\_TotalTime}}{\text{Manual\_TotalTime}}$$

$$\textbf{\% of False Positives} = 100 * \frac{\text{FP}}{\text{TP+PartialSyl+FN}}$$

$$\textbf{\% of Detected USVs} = 100 * \frac{\text{TP+PartialSyl}}{\text{TP+PartialSyl+FN}}$$
