# Supplementary material for: TrackUSF, a novel tool for automated ultrasonic vocalization analysis, reveals modified calls in a rat model of autism
Source: BMC Biol. 2022 Jul 12;20:159. doi: 10.1186/s12915-022-01299-y (PMC9277954; doi:10.1186/s12915-022-01299-y)
Supplement: Supplementary file 3 — Additional file 3. TrackUSF Manual. [file 12915_2022_1299_MOESM3_ESM.pdf]

TrackUSF

A software for analysis of ultra-Sonic vocalizations

TrackUSF is a software that analyze ultrasonic vocalizations in audio clips. It is doing so by analyzing ultra-sonic fragments of 6 ms duration within each clip and comparing these fragments between a collection of clips that were collected during an experiment. It is also useful for separating non-vocal strong sounds (such as noise from scratches of the cage floor) from the ultrasonic vocalizations. For more details, please refer to the paper:

Netser et al**. TrackUSF, a novel tool for unsupervised, automated and high-throughput analysis of ultrasonic vocalizations, reveals modified social communication in a rat model of autism**

The software was written in Matlab (2019a) and embedded the algorithm described in the paper above in a user-friendly graphical user interface (GUI). It was tested using both a standard HP-i7 computer (i7-4790 CPU @ 3.60 GHz, 8.0GB RAM, windows 7) and an HP workstation (HP Z6 G4 workstation, Xeon® Silver 4108 CPU @ 1.80GHz, 32GB RAM, windows 10).

Getting ready for using TrackUSFs:

This manual assumes that you have already installed Matlab (2019a or later) on your computer.

1. Download the software folder from GitHub (https://github.com/shainetser/TrackUSF) to your computer.
2. Start Matlab and choose the folder directory to be the one you downloaded TrackUSFs software to.
3. Make sure you have "Signal processing toolbox", "Statistics and machine learning toolbox" and "Parallel computing toolbox" installed in your computer. If not, use Matlab help and install them.

Important:

The example presented below was illustrated using two files - "BalbC_pair1_0200" and "C57_pair1_2100". For software examination please download these files from: MouseTube (https://mousetube.pasteur.fr/accueil.php, Protocol – "Week-long dyadic social interactions", User – "Yizhaq Goussha") and use a threshold of 2.7 as described along the manual. Upload each file into a different group (Named in the example "BalbCPair1" and "C57Pair1") using the "TrackUSF" GUI.

Using TrackUSF:

- Type in the command window "TrackUSF" and press enter.

The Graphical User Interface (GUI) will open:

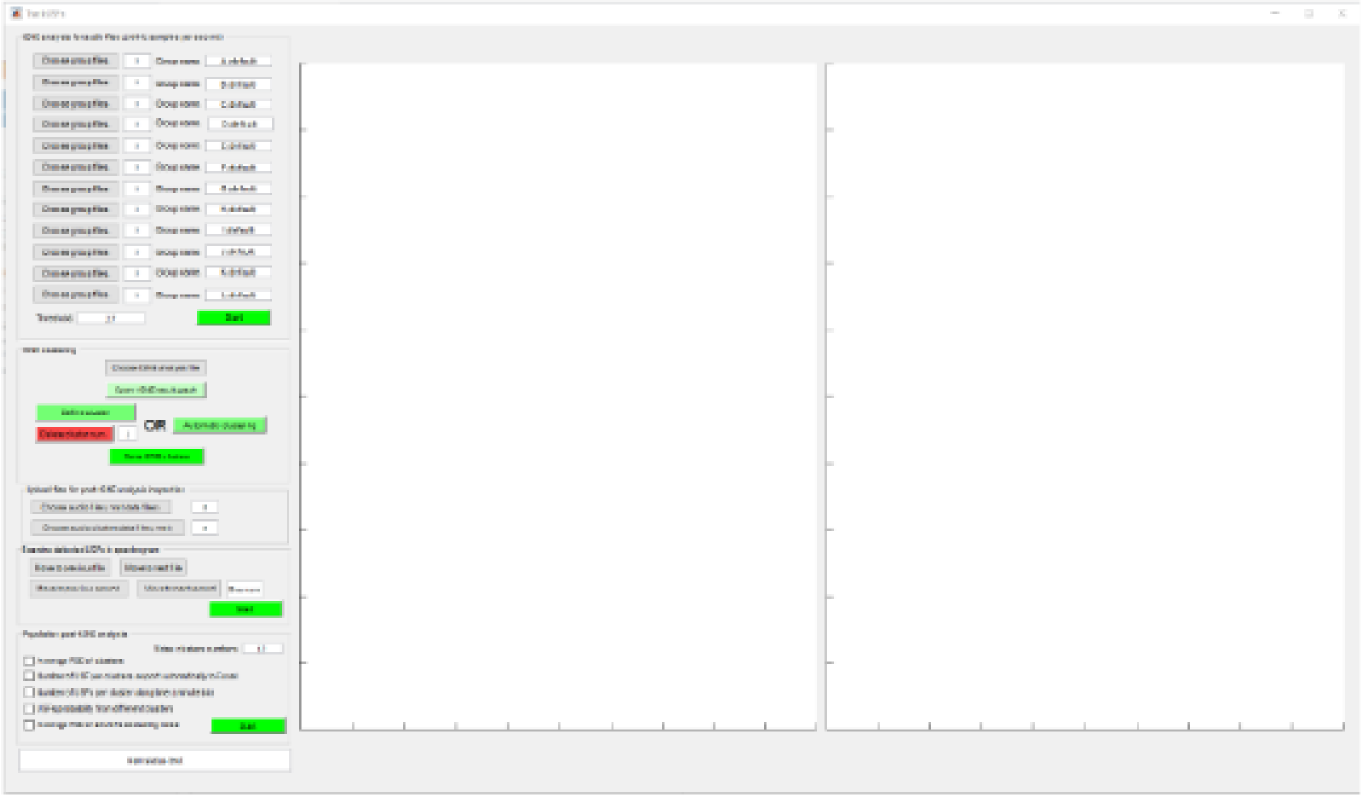

3D-t-SNE analysis:

- Using the "tSNE analysis for audio files" panel, upload all audio files ('WAV' format, sampled at 250 kHz) you would like to analyze for each group separately and name it accordingly (BalbC and C57 in the example presented here).

Set the threshold – for comparison of various threshold levels and the analysis look in Figure 3 of the paper mentioned above. It is recommended to start with 2.7 and then lower or lift it, according to the results and dataset.

When you are done, press "start" and you will be asked to choose a folder for saving the results in.

Important:

In certain conditions, Matlab may encounter a problem of memory limitations. Such an error is usually declared in the Matlab workspace as "out of memory" error. In such cases, please refer to MathWorks document:

https://www.mathworks.com/help/matlab/matlab_prog/resolving-out-of-memory-errors.html#brh72ex-54

A useful change is to "Increase System Swap Space" as detailed in the MathWorks document.

When the analysis is finished the software will create a 3D-tSNE graph of the results. For example:

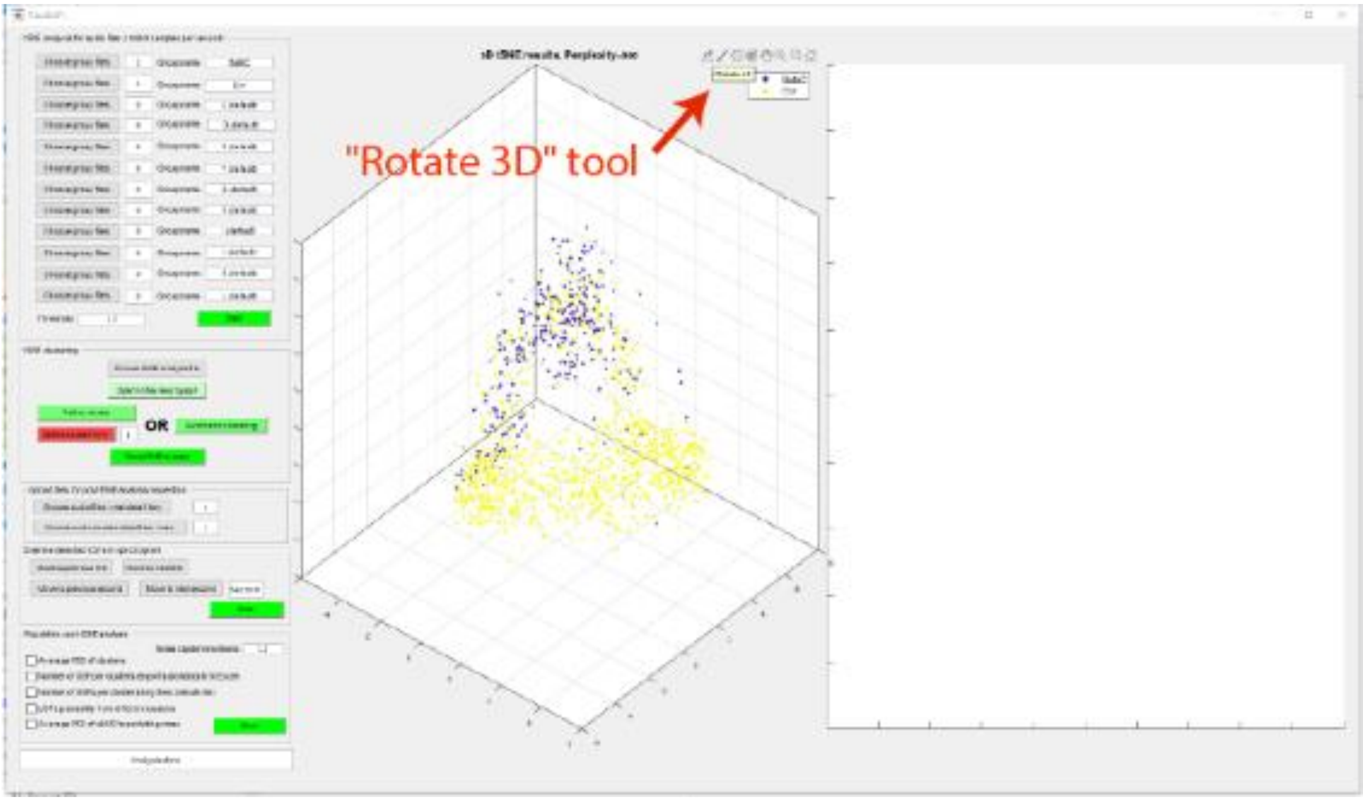

- The software also create a folder containing:

1) The tSNE results file (for example: '3D_tSNE_results 2020-05-19', '.mat' format).

2) Audio data files corresponding for each file loaded before. This files contains the audio data in a '.mat' format and will be used later on for generating a spectrogram for each file.

3) An MFCC data file (for example: 'Temp_MFCC_results 2020-05-19', '.mat' file).

- <u>Important:</u>

In case of a problem, a warning dialog will appear.

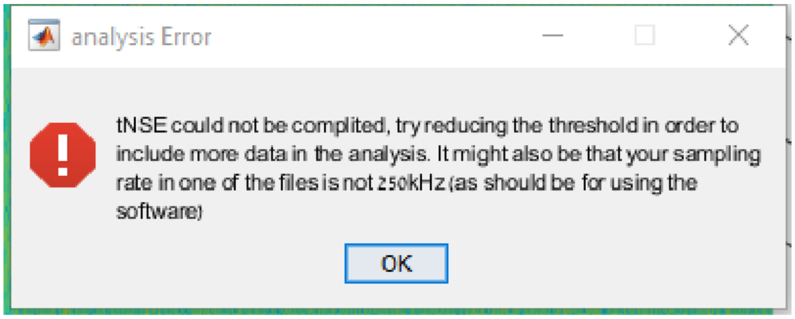

To solve it, try and reduce the threshold and repeat the analysis.

Clustering the fragments:

- In order to register the fragments obtained by the 3D-tSNE analysis into different clusters, move to the "tSNE clustering" panel and press the "choose tSNE analysis file" button.
  Choose the tSNE results file saved before. Now, press the "open tSNE result graph" button and the tSNE result figure will open in the left subplot of the GUI. (identical to the one previously displayed by the analysis). At this point you can rotate the 3D graph using Matlab "Rotate 3D" tool (See Figure 2). Try to visualize the optional clusters.
- At this point you may choose between manual definition of clusters and automatic clustering (using the function 'DBSCAN' of matlab) as follows.

Manual definition of clusters:

In order to associate a specific group of fragments (dots) with a specific cluster, press the "Define cluster" button and the "Brush/Select Data" tool of Matlab will start. At this point you can use it to mark a cluster of fragments by drawing a square around them, which will associate all fragments within the square with the given cluster.

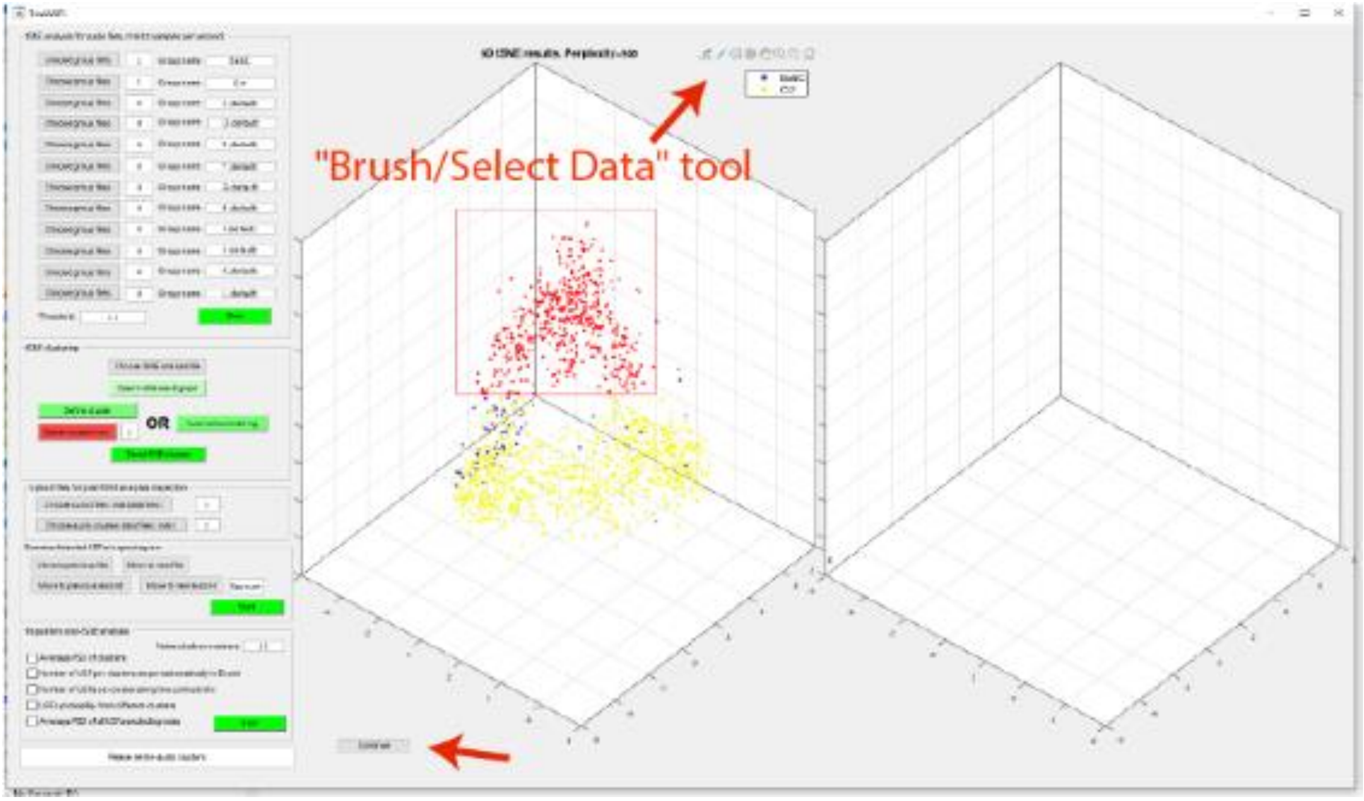

When done, press the "Continue" Button at the lower left corner of the figure.

The software then shows the clustered fragments in the right panel of the GUI and asks if you would like to save this cluster. Press 'Yes' or 'No', according to your satisfaction with the defined cluster.

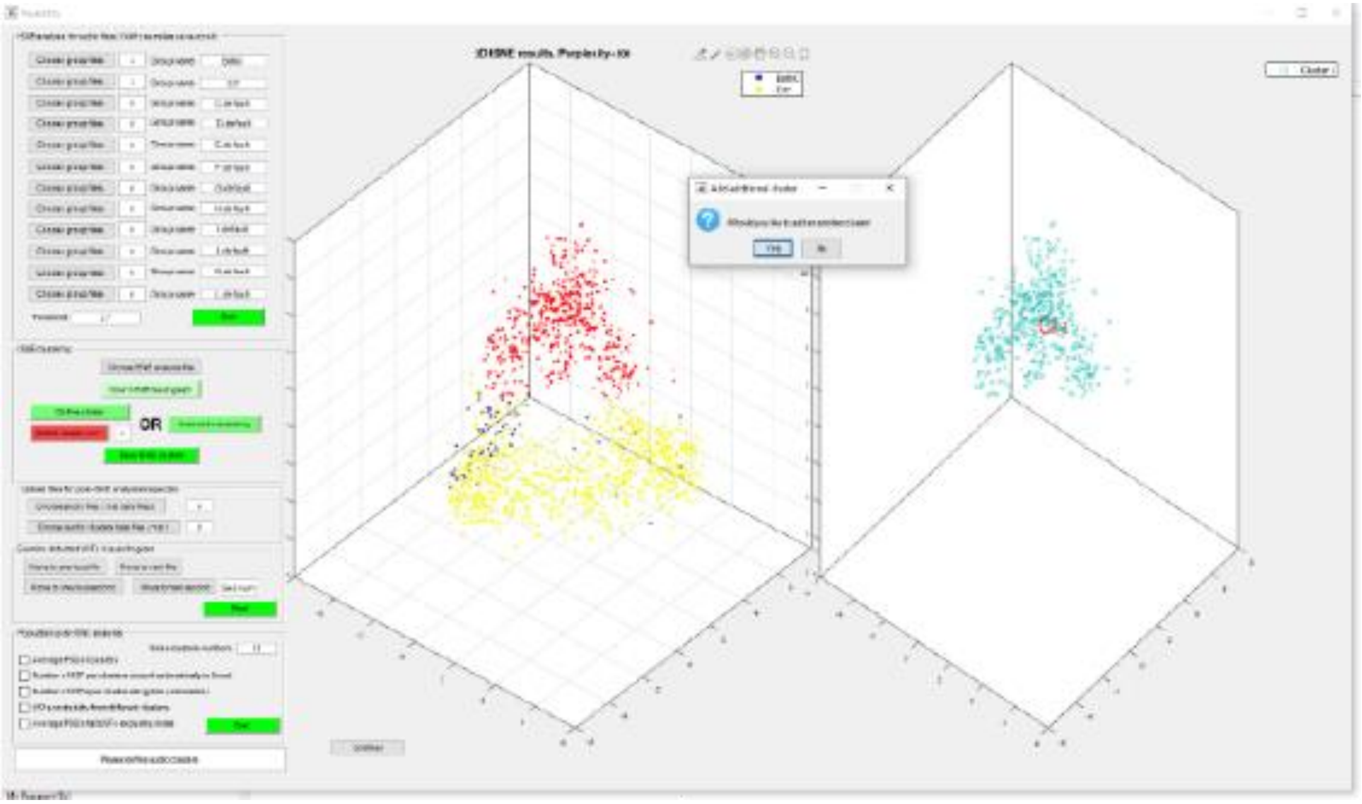

Then you will be asked if you would like to define an additional cluster. If you press 'Yes', you can immediately start marking an additional cluster, as the 'Brush/Select Data' tool is immediately active again. If you press 'No', you would later on be able to press the 'Define cluster' button again to mark more clusters.

In case you would like to delete a cluster, enter the cluster number you would like to delete and press the 'Delete cluster number' button. The cluster will then disappear from the right display.

When finished with defining clusters, press the 'Save tSNE vocalization groups' button and save the results under a specific folder.

This folder will contain:

1. Two tSNE plot figures. In the first, the dots are defined according to the experimental group, and in the second, according to the clusters they are registered to. Dots of the first figure that were not included in any of the defined clusters will not appear in the second figure.
2. For each of original WAV files loaded, the software will save one clusters data file ('.mat' format). This files contains the time points of detected fragments (from the beginning of the recorded file), according to the clusters they were registered to (Variable 'AllClustersForFile' if the file is uploaded to Matlab workspace). This can be used for further analysis and statistics if needed.

Important:

You can always shift between the '3D Rotate' tool and the 'Brush/Select Data' tool to get a better visualization of the cluster you would like to define, and only then define it.

Automatic clustering:

In order to automatically cluster the t-SNE result press the "Automatic clustering" button.

The process may take between few seconds to a minute and at the end of it the clustered fragments will appear in the right panel of the GUI.

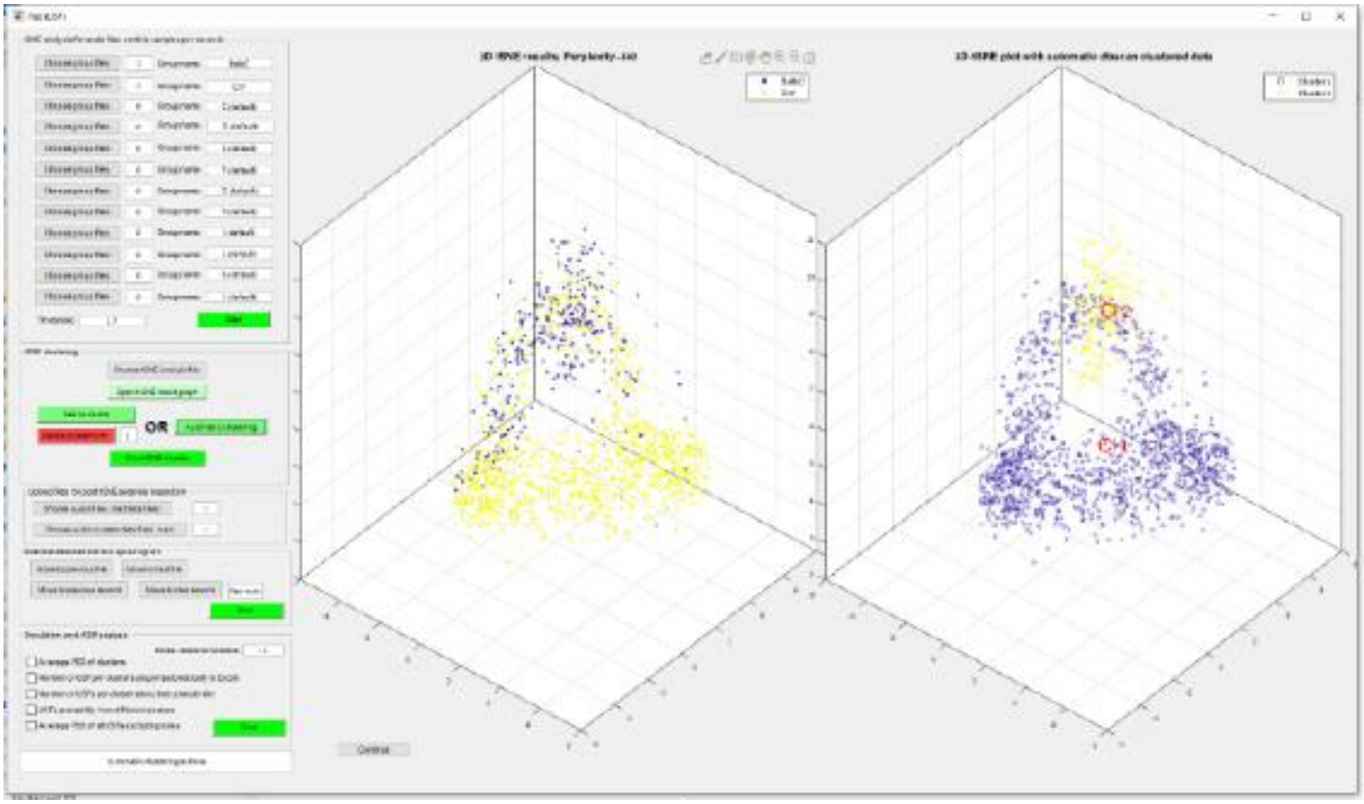

After automatic clustering, you may also add or remove clusters as explained in the "Manual definition of clusters" section above. Using this interactivity between manual and automatic clustering methods, you may cluster the fragments more accurately in case needed.

When finished with clusters definition, press the 'Save tSNE vocalization groups' button and save the results under a specific folder, as described above for manual clusters definition.

Important:

Automatic clustering is done using the function 'dbscan' in Matlab using the following parameters:

```
minpts=50;
epsilon=1.5;
dbscanIDX=dbscan([PopAudioAlone_tSNE_AnalysisData3D],epsilon,minpts);
```

Visualization of detected fragments:

For visualization of the detected fragments categorized to the different clusters on the audio clip spectrograms, use the 'Upload files for post-tSNE analysis inspection' and 'Examine detected USFs in spectrogram' panels. Press the 'Choose audio files (.mat data files)' button and choose one or several files generated in the first part of the analysis (obtained when running the 3D-tSNE analysis in the 'tSNE analysis for population of audio files' panel). Next, press the 'Choose audio clusters data files (.mat)' button and choose the corresponding files generated in the second part of the analysis (obtained when defining the clusters in the 'tSNE clustering analysis' panel). Keep choosing the clusters data files using the same order you chose the corresponding audio files. Pressing 'Start' in the 'Examine detected USFs in spectrogram' panel will result with the opening of a spectrogram in the left display of the GUI. This spectrogram is of the first second of the first file in the list. Each fragment will appear as the number of its cluster superimposed on the bottom of the spectrogram.
You can then move between files and seconds of file using the buttons:
'Move to next file', 'move to previous file', 'move to next second' or 'move to previous second'. You can also select a specific second by typing its number in the edit box and pressing enter. This way you can validate how well the software detected the various USVs and recognize the various types of USVs from which the various fragments were originated.

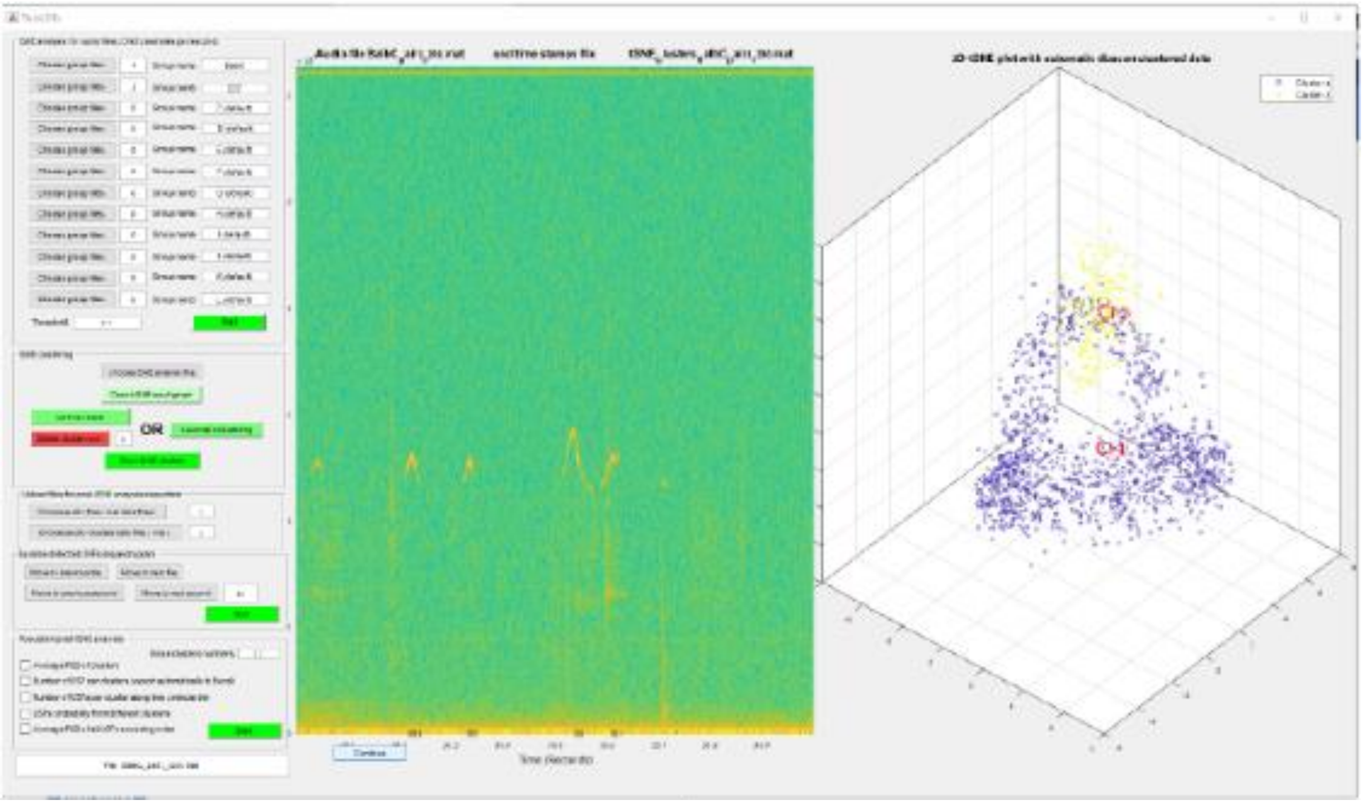

(In the example, detections of automatically detected cluster 1 fragments are marked at the bottom of the 30th second of the recorded file).

Important:

The two lists of files chosen above must be chosen in a corresponding manner, such that the audio data files correspond to the clusters data files.

Population analysis of detected fragments:

For a population analysis of the clustered fragments use the 'Population post-tSNE analysis' panel.
Choose from the various options listed in the panel by checking the relevant check boxes and then press the 'start' button. Defining specific clusters as "noise clusters" will exclude then from the results in the relevant graphs obtained (see main paper for examples)

*All variations of the software are deposited in GitHub under the following links:
https://github.com/shainetser/TrackUSF
